# Pharmacological bioactivity of *Ceratonia siliqua* pulp extract: in vitro screening and molecular docking analysis, implication of Keap-1/Nrf2/NF-ĸB pathway

**DOI:** 10.1038/s41598-023-39034-4

**Published:** 2023-07-27

**Authors:** Marwa El-Zeftawy, Doaa Ghareeb

**Affiliations:** 1 Biochemistry and Molecular Biology Department, Faculty of Veterinary Medicine, New Valley University, El-Kharga, New Valley Egypt; 2 Biological Screening and Preclinical Trial Lab, Biochemistry Department, Faculty of Science, Alexandria University, Alexandria, Egypt

**Keywords:** Biochemistry, Biological techniques, Plant sciences, Diseases

## Abstract

Inflammation is interfaced with various metabolic disorders. *Ceratonia siliqua* (CS) has a higher pharmaceutical purpose. The research aimed to investigate the biofunction of CS pulp aqueous extract (CS-PAE) with an emphasis on its integrated computational approaches as opposed to different specific receptors contributing to inflammation. The extract was assessed for its chemical and phenolic components via GC–MS, LC–MS, HPLC, and total phenolic and flavonoid content. In vitro*,* bioactivities and molecular docking were analyzed. Findings indicate that CS-PAE demonstrated higher scavenging activities of nitric oxide, 1,1-diphenyl-2-picrylhydrazyl radical, superoxide anion, hydrogen peroxide, and anti-lipid peroxidation (IC_50_ values were 5.29, 3.04, 0.63, 7.35 and 9.6 mg/dl, respectively). The extract revealed potent inhibition of RBCs hemolysis, acetylcholine esterase, monoamine oxidase-B, and α-glucosidase enzymes (IC_50_ was 13.44, 9.31, 2.45, and 1.5 mg/dl, respectively). The extract exhibited a cytotoxic effect against prostate cancer Pc3, liver cancer HepG2, colon cancer Caco2, and lung cancer A549 cell lines. Moreover, CS-PAE owned higher antiviral activity against virus A and some bacteria. When contrasting data from molecular docking, it was reported that both apigenin-7-glucoside and rutin in CS-PAE have a good affinity toward the Keap-1/Nrf2/ NF-ĸB pathway. In conclusion, CS-PAE showed promise in therapeutic activity in metabolic conditions.

## Introduction

Metabolic disorders are accompanied by inflammation, which inhibits kelch-like-enoyl-CoA hydratase associated protein-1 (Keap-1), leading to a decrease in the nuclear factor erythroid-2 related factor-2 (Nrf2) and increased nuclear factor kappa-B (NF-ĸB)^1^. Nrf2 is a principal leucine zipper transcription determinant regulated by Keap-1. Nrf2 organizes the expression of genes coding antioxidant enzymes like superoxide dismutase, catalase, and glutathione peroxidase, which preserve cellular stress^2^. NF-ĸB is a transcription factor of pro-inflammatory cytokine and one of the regulator genes related to apoptosis, angiogenesis, and inflammation. NF-ĸB is controlled in the cytoplasm by some protein suppressors such as NF-ĸB inhibitor alpha (IĸB-α) and NF-ĸB inhibitor beta (IĸB-ß), where they hinder its phosphorylation and translocation into the nucleus^3^.

Nowadays, various research articles focus on health-related bioactive isolated natural components, such as peptides derived from camel milk proteins that exhibit potential pharmacotherapy in chronic diseases^4^.

*Ceratonia siliqua* (CS), listed as one of the Angiosperms, family *Leguminosae*, genus *Certonia,* and *Ceratonia siliqua* L. was an accepted name^5^. The carob tree is a long-standing evergreen dioecious Mediterranean fruit tree that mostly grows in Spain, Italy, Portugal, Greece, Morocco, Turkey, Algeria, Tunisia, and Egypt. It is a member of the *Leguminosae* pedigree and the *Caesal Pinaceae* subfamily. CS tree produces fruits in the form of a pod that ranges in color from pale to dark brown, elongated, flat, straight, or mildly bent banana shape. CS pod comprises two main compartments, pulp representing 90% and seeds forming 10% of the overall fruit weight. The seeds are bounded with a close-fitting brown coat and comprise three layers, shell, endosperm, and embryo^6^.

The chemical composition and biological value of CS are varied based on the CS species, horticulture, environment, maturity stage, and different parts of the tree. The chemical profile of different CS parts was determined based on previously published investigations. CS leaves are rich in flavonoids, polyphenols, and fiber^7^. At the same time, the presence of anthocyanins like pro-anthocyanidins and ellagitannins, ascorbic acid (AA), and carotenoids such as lutein, lycopene, and carotene in CS pods give them potential biological effects^8^. CS seeds are distinguished by vitamin E vitamers and some organic acids like citric, oxalic, malic, and fumaric acids^9^. CS pulp is a plentiful supply of macro and micro elements, such as potassium, calcium, magnesium, sodium, copper (Cu^2^^+^), iron, manganese, and zinc^10^. Further, CS comprises several essential and non-essential amino acids, such as alanine, glycine, leucine, proline, valine, tyrosine, and phenylalanine, that contribute to protein biosynthesis, ion transport and membrane permeability^11^. CS contains water-insoluble polyphenols, multiple monosaccharides, disaccharides like glucose, fructose, maltose, and sucrose, and nutritional fibers like cellulose, hemicelluloses, and lignin^12^.

CS worth is involved in different branches of industry where the high sugar amount of CS makes it used as a natural sweetener and enters sweets manufacturing^13^. The powdered seedless pods are used as a cocoa or chocolate substitute as they are caffeine-free^14^. The CS pods' raw material is also used for syrup, crystallized sucrose, and wine production^15^. Further, Turhan, et al.^16^ reported ethanol production could be obtained from CS pod extract by *Saccharomyces cerevisiae* via batch fermentation. CS is used in gum preparation as a natural additive, flavoring agent, and stabilizer in multiple food products^17^. Recently, CS flour enhanced gluten-free products' nutritional values^18^. Also, adding CS extract to yogurt elevated its dietary standards^19^.

CS possesses many valuable biological activities that might have significance for the prohibition or treatment of some chronic conditions, like the protective effect of the gastrointestinal tract and antidiarrheal influence^20^. CS exhibited the ability to improve cardiovascular diseases resulting from some metabolic syndromes^21^. CS displayed a hepatoprotective effect against many toxic substances, such as alcohol and carbon tetrachloride^22^. Additionally, CS can treat chronic coughs, increase spermatogenesis and semen quality, and have a nephroprotective impact^23^.

In light of these facts, these work intentions are to fully explore Egyptian CS pulp aqueous extract (CS-PAE) through in vitro assessment of its numerous biological activities and analyzing the binding site of high compounds in CS-PAE by using docking approaches to confirm its therapeutic impacts.

## Materials and methods

### Chemicals and standards

1,1-diphenyl-2-picrylhydrazyl radical (DPPH), 3-(4,5-dimethylthiazol-2-yl)-2,5-diphenyltetrazolium bromide (MTT) and N-(1-naphthyl) ethylenediamine dihydrochloride were obtained from MP-Biomedicals-LLC (Germany), Bio-basic (Canada, INC) and Coinbrook Bucks (England), respectively. 5,5-dithio-bis-(2-nitrobenzoic acid) (DTNB), acetyl thiocholine iodide (ATCI), amphotericin, AA, acetonitrile, fetal bovine serum (FBS), gallic acid (GA), gentamicin, growth medium RPMI-1640, Mueller–Hinton agar, maintenance media (MEM), nicotinamide adenine dinucleotide hydride (NADH), nitrobluetetrazolium (NBT), penicillin, streptomycin, phenazine methosulfate (PMS), p-nitrophenyl glucopyranoside (pNPG), quercetin (QUR), Sabouraud's dextrose agar, sodium bicarbonate (NaHCO_3_), thiobarbituric acid (TBA) and sorafenib were got from Sigma-Aldrich (USA). Ammonium molybdate, trichloroacetic acid (TCA), absolute ethyl alcohol, acetic acid, aluminum chloride, benzylamine, dimethyl sulfoxide (DMSO), disodium hydrogen phosphate, ethyl acetate, ethyl ether, methyl alcohol, phosphoric acid, sodium dihydrogen phosphate, sodium hydroxide, sodium nitroprusside, sodium phosphate, anthrone, D-glucose and tris base were bought from (LOBA-Chemie). Potassium ferricyanide (K_3_Fe^3+^(CN)_6_), ferrous sulfate (FeSo_4_), ferric chloride (FeCl_2_), Folin-Ciocateu's reagent, hydrogen peroxide (H_2_O_2_), hydrochloric acid (HCL), potassium acetate, salicylic acid, sodium carbonate (Na_2_CO_3_), sodium chloride, sulfanilamide, sulfuric acid, vanillin, and trypsin were got from (Chemajet). However, isoflurane®, diclofenac potassium, donepezil, and metformin hydrochloride were gained from the pharmacy (Alexandria, Egypt).

### Plant material

The fully developed CS pods were gathered from the native store in Alexandria governorate, Egypt. The gathering of CS pods complied with relevant institutional, national, and international guidelines and legislation.

The water extraction method with some modifications described by Rtibi et al.^24^ was used for the powdered pulp. The supernatant was gathered and sieved across a sterile filter (0.5 mm mesh size). The concentrated aqueous extract was obtained by solvent evaporation under lower compression at 40–45 °C using a rotary evaporator. After that, the extract was freeze-dried by lyophilizer for 99 h (hs.) with a pressure of 18 mT at the condensing temperature of 82.8 °C to obtain a crude extract form. This crude form was subjected to ultra-sonic sonicate for 72 h to remove any moisture content. The obtained crude form (102.7 g/500 g of CS pulp powder) was stored at − 80 °C until use.

All in vitro assays were carried out on three CS-PAE samples and standard drugs on three consecutive days. The dose of CS-PAE in the current study was determined according to the pilot study, where (0.8, 1, 2, 4, 6, 8, and 10 mg/ml) was used for antioxidant and anti-inflammatory activities and enzyme inhibition assays and (78.12, 156.25, 312.5, 625, 1250, 2500, 5000, and 10,000 µg/ml) was utilized for anticancer and antiviral cytotoxicity.

### Experimental rats

Adult male Wistar rats of Bwt of 200–250 g and 2–3 months old were obtained from the Pharmaceutical and Fermentation Industries Development Center-SRTA city, Alexandria, Egypt. Rats were housed for a week to acclimatize in exact environmental situations of relative humidity (20%), temperature (22 ± 1 °C), and 12 h/12 h light/dark cycle. Food and water (H_2_O) were always supplied.

The management of rats was performed under animal welfare guidelines after fulfilling the requirements of the Committee implementation for the Institutional Animal Care and Use Committees (IACUCs)/IACUC#**65-3H-9022**. All attempts were made to lessen the rats' pain.

### Identification of CS-PAE components

Gas chromatography–Mass spectrum (GC–MS) was carried out to scan the chemical structure of CS-PAE according to Abd El kareem et al.^25^ with some modification, using a Trace GC1300-ISQ mass spectrometer with a direct capillary column TG–5MS (30 m × 0.25 mm × 0.25 µm film thickness). The column oven temperature was grasped at 60 °C, then highest by 5 °C/min to 200 °C and held for 2 min. Then increase to the eventual temperature of 300 °C by 20 °C/min and hold for 2 min. The injector and MS transfer line temperatures, were conserved at 250 °C and 260 °C, respectively; helium was used as a carrier gas at a fixed influx rate of 1 ml/min. The dissolvent delay was three minutes, and 1 µl of diluted sample was injected automatically using Autosampler AS1300 coupled with GC in the split mode. Electron impact mass spectra were gathered at 70 eV ionization voltages over the m/z 50–650 range in whole scan mode. The ion exporter temperature was adjusted at 250 °C. The components were specified by comparison of their retention periods and mass spectra with those of WILEY 09 and NIST 11 mass spectral databases.

Further, the phenolic components in CS-PAE were recognized and quantified by high-performance liquid chromatography (HPLC). Firstly, phenolic acids in the sample were extracted^26^, then filtered using an Acro-disc syringe filter (0.45 µm size) (Gelman Laboratory, MI), and only 50 μL of the sample was injected for the HPLC test. HPLC analysis was done by Agilent Technologies 1100 series liquid chromatograph containing an auto sampler and a diode-array sensor. The analytical column was Eclipse XDB-C18 (150 × 4.6 µm; 5 µm) with a C18 guard column (Phenomenex, Torrance, CA). The mobile phase included solvent A, acetonitrile, and solvent B, 2% acetic acid in H_2_O. Additionally, the flowing rate was conserved at 0.8 ml/min. for a total run time of 70 min. and the gradient scheme was as follows, 100% B to 85% B in 30 min, 85% B to 50% B in 20 min, 50% B to 0% B in 5 min and 0% B to 100% B in 5 min At 280 and 320 nm, sample peaks were observed for benzoic acid and cinnamic acid derivatives, respectively. The obtained peaks were recognized by identical retention times and ultra violet (UV) spectra; then compared with the references^27^.

Additionally, liquid chromatography–mass spectrometry (LC–MS) analysis of CS-PAE was executed. LC–MS was based on the separation of specific extract components by LC, followed by the creation and detection of the charged ions by MS. LC–MS was performed by the LC-2040 instrument. The chromatographic system comprised a data acquisition, an LC-2040 pump, and an LC-2040 autosampler. The mobile phase used for analysis was formed from twice phases; A and B. Phase A was formed from an ultrapure H_2_O, while phase B comprised acetonitrile. Electrospray ionization (ESI) included negative and positive modes. ESI was operated at the next conditions: the interface temperature was 300 °C, the desolvation temperature was 526 °C, the DL temperature was 250 °C, the nebulizing gas flow was 3.0 l/min., the heating gas flow was 10.0 l/min., heat block temperature was 400 °C and drying gas flow was 10.0 l/min. Compounds were assigned in CS-PAE by comparing their molecular weights to reference literature.

### Measurement of total phenolic and flavonoid contents in CS-PAE

With some modifications, the Folin-Ciocalteu technique evaluated total phenolic content (TPC) according to Ohadoma, Akuodor, Amazu and Michael^28^, and the TPC was calculated from the standard curve of GA. The findings were stated as microgram GA equivalent/milligram CS-PAE.

Also, total flavonoid content (TFC) was estimated following the aluminum chloride colorimetric procedure^29^. The flavonoid quantity in the CS-PAE sample was estimated from the calibration graph of standard QUR. The outcomes were presented as microgram QUR equivalent/milligram CS-PAE.

### Assessment of total carbohydrate, lipid, and protein contents in CS-PAE

The whole carbohydrate constituent was analyzed depending on the previously described anthrone method, where the extracted sample was digested by 2.5 N HCL and neutralized by Na_2_CO_3_ and centrifugation. D-glucose served as a standard. The total carbohydrate content was assessed at 630 nm, calculated through the standard curve, and expressed as microgram D-glucose equivalent/ milligram CS-PAE extract^30^.

The slightly modified sulfo-phospho-vanillin technique described by Mishra et al.^31^ was used to reveal the total lipid content of a sample, and palmitic acid acted as a lipid standard.

Further, the Lowry method analyzed the extract's total protein content by using phosphomolybdic-tungstic mixed acid, which was the dynamic component of the folin-phenol reagent of folin and ciocalteu in the reaction. These combined acids in the foiln-ciocalteu preparation were the last chromogen. Proteins in the sample affected the reduction of mixed acids by losing one, two, or three oxygen atoms from tungstic acid and-/or molybdic acid and yielded several reduced species. Further presence of Cu^2+^ in one reagent used in the reaction chelated the peptide structure of the protein in the sample, where it facilitated electron transmit to the blended acid chromogen in the area of a functional amino acid group by raising the sensitivity to protein^32^.

### Analyzing the antioxidant activity of CS-PAE

#### Total antioxidant capacity (TAC) and ferric-reducing power (FRP)

TAC was established on the reaction between sodium phosphate and ammonium molybdate at acidic pH media to form phosphate-molybdate (VI), then phosphate-molybdate (VI) in the presence of CS-PAE (sample), AA (standard), or 10% DMSO (vehicle) was reduced to phosphate-molybdate (V). The technique was implemented based on a prior investigation by Tyagi et al.^33^ with slight modification. TAC in the sample was computed via plotting reference chart and described as mg/ml CS-PAE.

FRP assay was performed via the potassium ferrocyanide-ferric chloride method that relied on the reaction of the tested substance, which has antioxidant properties with K_3_Fe^3+^(CN)_6_ to produce potassium ferrocyanide (K_4_Fe^2+^(CN)_6_) that reacts with FeCl_2_ to form a ferric-ferrous complex. This reaction generates a very strong blue color. The intensity of the produced color positively correlates to the examined substance's reduction power^34^. The reduction capability of CS-PAE was computed using the AA calibrating curve and formulated as mg/ml.

#### Antiradical assays

The inhibitory activity of CS-PAE against the radicals was determined by nitric oxide (NO), DPPH, superoxide anion, and H_2_O_2_ radicals scavenging activity using an enzyme-linked immunosorbent assay (ELISA) reader. The percentages of radicals scavenging activity were calculated by using the following formula: % of radical scavenging activity = [(A_BL_–A_T_)/ A_BL_]*100, where A_BL_ is the blank absorbance, and A_T_ is the CS-PAE or AA absorbance. Also, the inhibitory concentration (IC_50_) that inhibited 50% of analyzed radicals of the CS-PAE and AA was calculated by a graph plotting scavenging activity against concentration.

NO scavenging activity was performed in line with the modified procedure of Marques et al.^35^, who used the *Griess* reaction technique. In addition, the current research analyzed the capability of CS-PAE to scavenge DPPH. DPPH molecule is considered one of the stable free radicals due to the spare electron on the molecule, which generates deep violet color in ethanolic or methanolic solutions. The scavenging performance of examined CS-PAE depended on the transformation of the DPPH radical to the matching hydrazine via the contribution of a proton to form the reduced DPPH, so the solution color became yellow instead of violet^36^.

Further, the capability of CS-PAE to salvage the superoxide anion radical (SAR) was measured following the method of Alagumanivasagam^37^ with slight modifications. The assay was chiefly based on generating SAR in the PMS-NADH system through NADH oxidation and measured via NBT reduction.

The H_2_O_2_ radical scavenging activity assay was performed on the bases of the H_2_O_2_ reaction with ferrous ions (Fe^2+^) at low pH that leads to oxidation of Fe^2+^ to ferric ion (Fe^3+^), and H_2_O_2_ was divided into hydroxide ions (^−^OH) and hydroxyl radicals (^·^OH)^38^. The current experiment was executed in triplicate in the existence of hydroxylated salicylic acid, forming 2,3- and 2,5-dihydroxybenzoic acids. Hence, to examine the scavenging effect of CS-PAE toward H_2_O_2_, it must first generate H_2_O_2_ in the reaction using salicylic acid, FeSO_4_, and H_2_O_2_. Briefly, the sample and standard were prepared by mixing 50 µl of every CS-PAE or AA concentration with 50 µl salicylic acid (9 mmol/l), 50 µl FeSO_4_ (9 mmol/l), and 50 µl H_2_O_2_ (9 mmol/l), and the absorbance was read at 405 nm after 60 min incubation at 37 °C. The blank and sample blank was prepared in the same method; however, ddH_2_O was added in blank instead of CS-PAE and in sample blank instead of H_2_O_2_.

#### Anti-lipid peroxidation

The procedure of^39^ determined the inhibition frequency of CS-PAE against lipid peroxidation with minor modifications. The assay mainly depends on using a source of polyunsaturated fatty acids like liver or brain homogenate and assessing the extract capacity to suppress the peroxidation. In short, three healthy adult male *Wistar* rats were sacrificed under anesthesia by isoflurane®, and 10% of liver homogenate was produced in ice-cold phosphate buffer saline (PBS) (0.1M, pH 7.4). After that, 1 ml of each CS-PAE (sample) and AA (standard) dosage and ddH_2_O (blank) were added to 1 ml of clear supernatant of prepared liver homogenate and 100 µl of FeSO_4_ (15 mM) in tubes. The tubes were incubated at 37 °C for 30 min. Then 100 µl of such reaction mixture was added to 1.5 ml of 10% TCA, and the tubes were re-incubated for another 10 min. at room temperature. After that, the tubes were centrifuged at 4000 rpm for 10 min. using a table centrifuge. Finally, 0.5 ml of 0.67% TBA was added to 1 ml of collected supernatant, heating the content of tubes in a boiling water bath at 95 °C for 30 min. The absorbance was determined at 535 nm via a spectrophotometer versus the blank. The inhibition ratio (%) was obtained, and the extract concentration generating 50% lipid peroxidation inhibition (IC_50_) was assessed.

### Anti-inflammatory effects of CS-PAE

The red blood corpuscles (RBCs) membrane stabilizing technique was done to estimate the anti-inflammatory activity of CS-PAE. The method is based on the power of non-steroidal anti-inflammatory therapies to inhibit the lysosomal enzymes generated during inflammation or stabilize the lysosomal membrane. It was noticed that using RBCs in the experiment was like lysosomal membrane composition, and the prohibition of hypotonicity-induced RBCs membrane lysis is regarded as a sign of the anti-inflammatory effects of the CS-PAE. The assay was done according to the modified method^40^, and the source of whole blood in the experiment was male rats, and diclofenac potassium (50 µg/ml) was used as standard. The inhibition percentage of RBCs hemolysis and the IC_50_ value was calculated.

### Neurologic effects of CS-PAE

The neurological effect of the extract was estimated by measuring the efficacy of monoaminoxidase-B (MAO-B) and acetylcholinesterase (AchE) enzymes. Brain homogenate of rats was required to perform this experiment as a source of both tested enzymes. The results of CS-PAE were compared to the standard drug, donepezil.

The capability of CS-PAE to increase or decrease MAO-B activity was examined by the kinetic method adapted from Akomolafe et al.^41^ with modifications using a UV spectrophotometer at 250 nm, and benzylamine was used as a substrate for the MAO-B enzyme. This equation calculated the enzyme activity: MAO-B activity (U/I) = [(∆A*Total reaction volume*1000)/ (32.2*Sample volume*0.5)] where ∆A is the difference of absorbance after 90 and 30 s. Also, the capability of the sample and standard to obstruct the MAO-B was calculated and expressed as a percentage.

Moreover, as previously published by Attanayake and Jayatilaka^42^ with only minor modifications, the cholinergic activity of CS-PAE has been assessed by measuring the AchE inhibitory percentage. The method mostly relied on the extract's ability to inhibit the AchE responsible for acetylcholine hydrolysis, which leads to a disturbance in nervous signal transmission and causes multiple neurodegenerative diseases.

Concisely, in 96 well ELISA plate, 20 µl of CS-PAE (sample) or donepezil (standard) different concentrations or ddH_2_O (blank) was added to 130 µl of PBS (0.1M, pH 7.4), and 10 µl of rat's brain homogenate. The sample blank was like the previous reagent mixture; however, 10 µl of PBS (0.1M, pH 7.4) was used instead of the rat's brain homogenate. The plate's content was mixed via shaker and incubated at 37 °C for 30 min. After that, 5 µl of ATCI (75 mM in ddH_2_O) was put into each plate's wells and left for 15 min. at 37 °C. In the end, 10 µl of DTNB, known as *Ellman's* reagent (0.32 M in phosphate buffer pH 7.4 contains 15 mg NaHCO_3_), was put over the plate content, and the transmission density was read at 405 nm directly after 5 min. by ELISA reader. The following formula calculated the percentage of AchE inhibition: Inhibitory rate (%) = [(A_BL_–A_T_)/ A_BL_]*100, where A_BL_ is the blank absorbance, and A_T_ is the CS-PAE or donepezil absorbance. Also, the CS-PAE and donepezil dose that was wanted to obstruct AChE activity by 50% (IC_50_) was determined.

### Antidiabetic effects of CS-PAE

The antidiabetic influence of CS-PAE was evaluated via α-glucosidase inhibition activity assay. The procedure relied on the capability of the tested extract to delay the function of an α-glucosidase enzyme in the hydrolysis of disaccharides into monosaccharides. The experiment was conducted in triplicate using the appropriate methodology stated by Alara et al.^43^, and rats' liver homogenate was used as a source of enzyme, metformin hydrochloride was the standard, and pNPG (5 mM) was the substrate. The α-glucosidase inhibitory rate was demonstrated by measuring the yellow-colored para-nitrophenol liberated from pNPG at 400 nm. The data were represented as a percentage. The IC_50_ of examined CS-PAE, the reference drug, and the percentage of α-glucosidase inhibitory activity were calculated.

### Anticancer effects of CS-PAE

#### Tumor cell lines and cell culture

Prostate cancer Pc3, liver cancer HepG2, colon cancer Caco2, and lung cancer A549 cell lines were attained from Vacsera Cell Culture Unit, Dokky, Giza, Egypt. The cells were preserved in a solid cap flask containing the growth medium RPMI-1640 supplemented with 10% heat-inactivated FBS, 1% penicillin–streptomycin, and 0.2% NaHCO_3_ in completely humid circumstances of 95% air and 5% carbon dioxide (CO_2_) at 37 °C.

#### Cytotoxicity assay

Cellular cytotoxicity was analyzed by the MTT reduction colorimetric method. The assay depended on the ability of mitochondrial succinate dehydrogenase enzymes in living cells to lessen the yellow water-soluble substrate, MTT, into an insoluble, colored formazan output measured spectrophotometrically. The assay was carried out according to^44^. At first, the growth medium RPMI-1640 in the solid cap flasks was removed, and the flasks that contained adhered cells were washed twice with PBS. Then, 2 ml of trypsin was added to the flasks for 3 min at 37 °C in the CO_2_ incubator. Later incubation, the flasks were examined under an inverted microscope to confirm the complete cell detachment. Then each type of cancer cell in the flask was resuspended in 10 ml of growth medium.

Subsequently, each tested tumor cell was inoculated in a separate 96-well tissue culture plate with a range of 1 × 10^5^ cells/ml (100 µl/well) and incubated at 37 °C for 24 h. to create a full monolayer sheet. Following the incubation time and establishing a confluent sheet of cells in the plate, the growth medium was withdrawn from the plate wells, and the cell monolayer was twice washed with washing media (PBS, 0.1M pH 7.4).

Two-fold dilutions of CS-PAE (78.12, 156.25, 312.5, 625, 1250, 2500, 5000, and 10,000 µg/ml) were made in a maintenance RPMI medium with 2% FBS to be used in the assay. After sample preparation, 100 µl of each dilution was applied to the wells of each cell plate (sample), and in each plate, three wells received only MEM (control). Sorafenib was used as a standard antitumor drug with the same CS-PAE doses, dissolved in DMSO, and added in standard plate wells (standard)^45^. The plates were incubated at 37 °C for 24 h. The next day, they were examined under an inverted microscope. Cells were examined for toxicity-related physical symptoms such as partial or complete loss of the monolayer, rounding, shrinkage, or cell granulation.

Afterward, 35 µl of MTT solution (5 mg/ml in PBS) was added to each well, and the plate's content was thoroughly mixed on a shaking table at 150 rpm for 5 min. The plates were incubated in a CO_2_ incubator at 37 °C, and 5% CO_2_ was supplied for three hours to allow the metabolization of MTT.

After the incubation, the media was thrown away, and plates were dried to eliminate any residues. The reaction was obstructed by applying 120 µl of DMSO to all wells to resuspend the formed formazan, MTT metabolic product. Plates were shacked again on a shaking table at 150 rpm for 5 min. to combine the formazan into the solvent thoroughly. The developed red color was read on a Micro ELISA reader using two wave lengths (570 and 620 nm), and the number of cells should be closely connected with optical density. The median inhibitory concentration needed to suppress 50% of cell viability (IC_50_) was illustrated graphically and calculated.

The impact of CS-PAE and sorafenib on the propagation of each tumor cell was represented as the % cell viability, using the succeeding formula: Cell viability (%) = (A_T_–A_C_)*100, where A_T_ is the mean of each CS-PAE or sorafenib concentration optical density and A_C_ is the mean of control optical density. Also, the toxicity % was calculated by the following equation: Cell toxicity (%) = 100—cell viability %.

### Antiviral effects of CS-PAE

#### Estimation of the maximum non-toxic concentration (MNTC) of CS-PAE on VERO cell

The VERO kidney epithelial cell line (*Cercopithecus aethiops*), ATCC CCL-81, was purchased from the Faculty of Medicine for girls, Microbiology Department, Azhar University, Egypt. After the confluent sheet of the VERO cell was created, the growth medium was poured from 96-well microtiter plates, and the cell monolayer was washed twofold with a washing media. Different CS-PAE samples (78.12, 156.25, 312.5, 625, 1250, 2500, 5000, and 10,000 µg/ml) were prepared, and double-fold dilutions were done in MEM. In the adhesive VERO cell microtiter plate, 0.1 ml of every sample dilution was added to numerous wells, leaving three wells as control, receiving only the MEM. After that, the plate was incubated at 37 °C and inspected regularly for up to 48 hs. The physical appearance of any cell toxicity was examined, such as partial or whole lack of the monolayer, rounding, shrinkage, or cell granulation. After two days, 20 µl of MTT solution (5 mg/ml in PBS) was added to each plate well. The plate content was thoroughly mixed using the shaker at 150 rpm for 5 min. to ensure a good combination of the MTT in the media. Then, the microtiter plates were incubated in a CO_2_ incubator (37 °C, 5% CO_2_) for 1–5 h. to permit the MTT to be metabolized. Subsequently incubated and discarded the extra media, 200 µl of DMSO was added to all wells to the resuspend formazan (MTT metabolic product). The plates had been set on a shaker at 150 rpm for 5 min. again, to thoroughly mix the formazan into the solvent. Lastly, the optical density of plate content was read at 560 nm and subtracted background at 620 nm via ELISA reader. The MNTC of CS-PAE was determined and was used for further antiviral study. Also, the 50% cytotoxic concentration (CC_50_), the CS-PAE concentration that wanted to reduce the cell viability by half, was calculated^46^.

#### Antiviral assay

The antiviral activity was estimated using a slightly modified method^47^. Initially, VERO cells were seeded at 10,000 cells in 200 µl media per well in a 96-tissue culture plate and incubated overnight in a CO_2_ incubator. The MNTC of CS-PAE protective effect against viral infection was examined. Three types of virus suspension got from the Medicine for girls Faculty, Microbiology Department, Azhar University, Egypt, were used in our study. They were hepatitis A virus (HAV), Coxsackievirus B4 (CoxB4), and herpes simplex virus 1 (HSV1).

First, the virus suspension of each individual studied virus, HAV, CoxB4, and HSV1, combined with MNTC of CS-PAE, was prepared (1:1 v/v) and incubated for an hour before the infection trial. In tissue culture plate wells, add 100 µl of each type of virus suspension alone (in control virus wells), 100 µl of each type of prepared virus suspension with MNTC of CS-PAE (in CS-PAE protective wells), and 100 µl of MNTC of CS-PAE alone (in control wells). All infected and control cell plates were put on the shaking table at 150 rpm for 5 min. Then, they were incubated in a CO_2_ incubator for 1-day. On the second day, 20 µl of MTT solution (5 mg/ml in PBS) was added to all plate wells. The plate's content was well mixed and incubated in a CO_2_ incubator for five hours. Then, 200 µl of DMSO was added to the plates to stop the reaction. The optical density was read at 560 nm, and the background was subtracted at 620 nm using an ELISA reader.

The antiviral impact of CS-PAE MNTC on HAV, CoxB4, and HSV1 activity was calculated alone and stated as %, using the following equation: Antiviral activity (%) = 100–[(A_T_–A_C_)*100], where A_T_ is the optical density mean of each MNTC of CS-PAE against the examined virus, and the A_C_ is the optical density mean of control.

### Antimicrobial effects of CS-PAE

Antimicrobial activity was achieved by the agar well diffusion technique, based on the capability of CS-PAE to prevent bacterial or fungal growth via noticing its diffusion rate in a solid nutrient medium^48^. The microbial strains used in this experiment were from the Faculty of Sciences, Al-Azhar University, Egypt. We selected in our study Gram-positive bacteria (*Bacillus subtilis* and *Staphylococcus aureus*), Gram-negative bacteria (*Escherichia coli* and *Klebsiella pneumonia*), and fungal cultures (*Candida albicans and Aspergillus flavus)*. The experiment was conducted in 3 replicates.

The tested microorganisms (Mos) were seeded into a specific medium by adding 0.5 ml containing 1.8 × 10^8^ CFU/ml of 24- and 48-h (h) new cultures for bacteria and fungi, respectively, to 20 ml sterile melted Mueller–Hinton agar (for bacteria) and Sabouraud's dextrose agar (for fungi) at 45 °C. After cooling, the medium containing Mos was poured into sterile Petri plates. Following the hardening of all plates, 6 mm diameter wells were done by a sterilized perforator. The wells in Mueller–Hinton agar plates were impregnated with 50 µl of 50 mg/ml CS-PAE (sample) and 10 mg/ml gentamicin (standard antibacterial agent). The wells in Sabouraud's dextrose agar plates were filled with 50 mg/ml CS-PAE (sample) and 5 mg/ml amphotericin (standard antifungal agent). The bacterial cultures were incubated at 37 °C for 24 h, while another fungal culture was incubated at 25–30 °C for 3 to 7 days. The antimicrobial potency of the CS-PAE and standards was determined by measuring the diameter of the inhibition zone surrounding the wells, and it was expressed in mm. It was also considered positive only when the inhibition zone was more than 6 mm.

### Molecular docking studies

The computational (in silico) technique has been widely used as an efficient instrument for virtual biological screening. This method evaluated the biological activities and estimated affinities of natural products, synthetic compounds, and semisynthetic molecules^49^.

In this study, fifteen natural metabolites were evaluated against the NF-ĸB and Keap-1/Nrf2 pathways to show the affinity of our compounds against these target sites. At first, proteins were downloaded from the protein data bank (proteins Id: 5T8P and 5CGJ). All proteins and the natural metabolites were prepared, and the MMFF94 force field lessened energy. The molecular docking was done, twenty poses were generated, then the best orientations were captured, and affinity scores and root mean square displacement (RMSD) values were collected.

### Statistical analysis

Statistical analysis was executed using the SPSS program (Version 25, ANOVA one-way test). Data are displayed as mean ± SE, and the *p*-value < 0.05 was revealed to be statistically significant.

### Ethical approval

The study was conducted following ARRIVE guidelines (https://arriveguidelines.org) and approved by the Ethics Committee application for the Institutional Animal Care and Use Committees (IACUCs)/IACUC#**65-3H-9022** of Pharmaceutical and Fermentation Industries Development Center-SRTA city, Alexandria; Egypt.

## Results and discussion

### Identification of CS-PAE components

**Figure 1:**
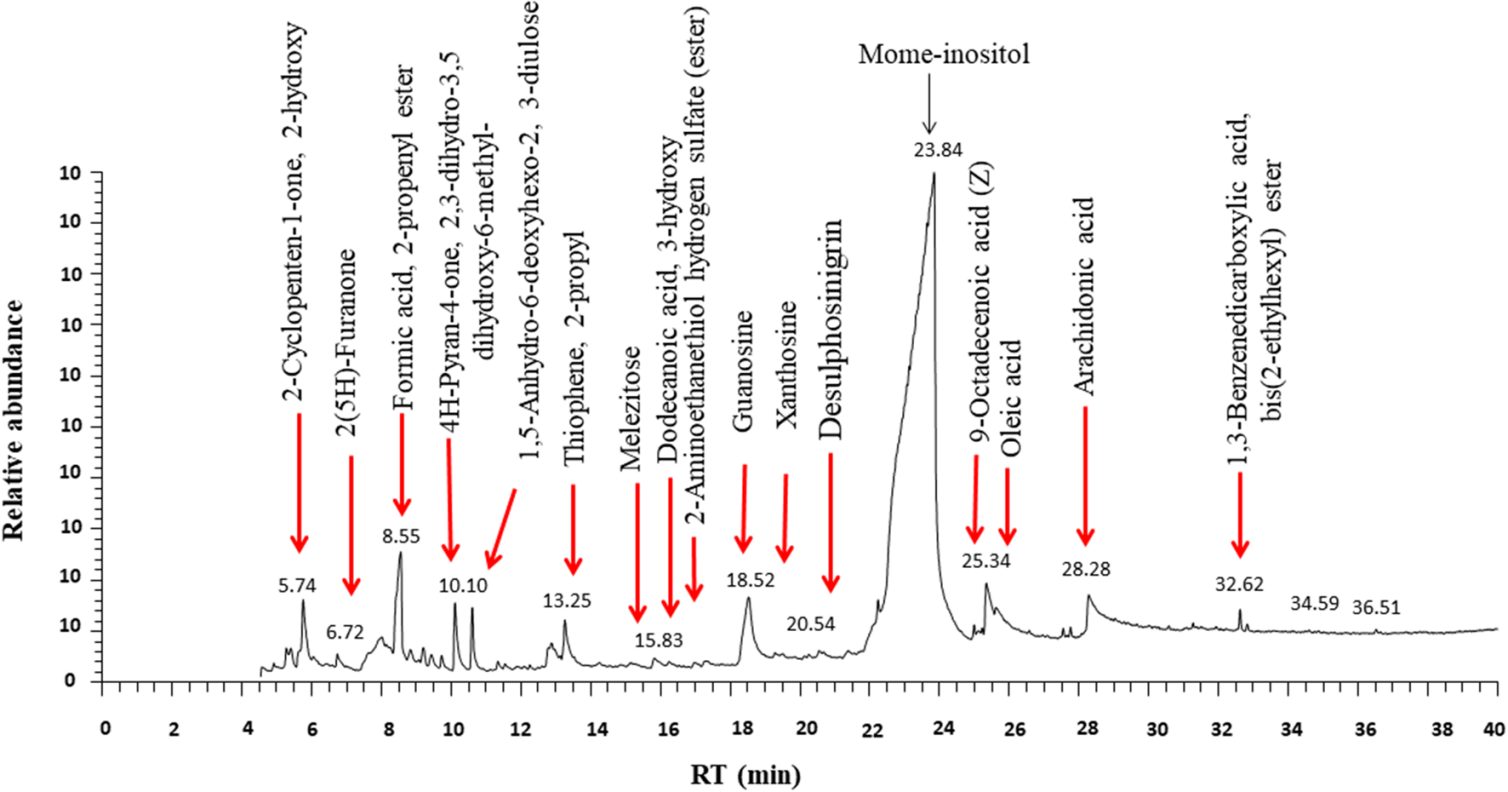
GC chromatogram of CS-PAE.

**Figure 2:**
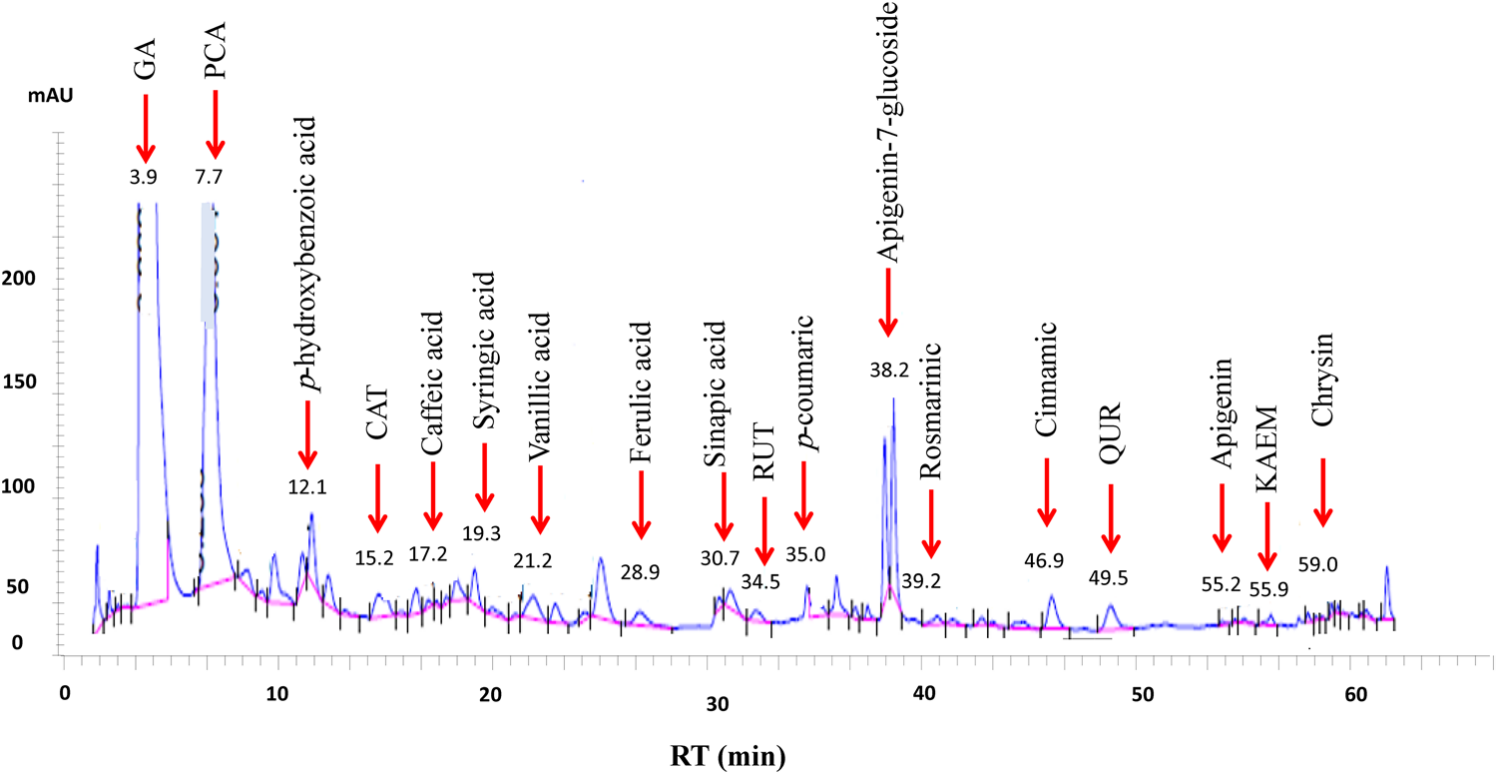
HPLC chromatograms of phenolic compounds in CS-PAE.

**Figure 3:**
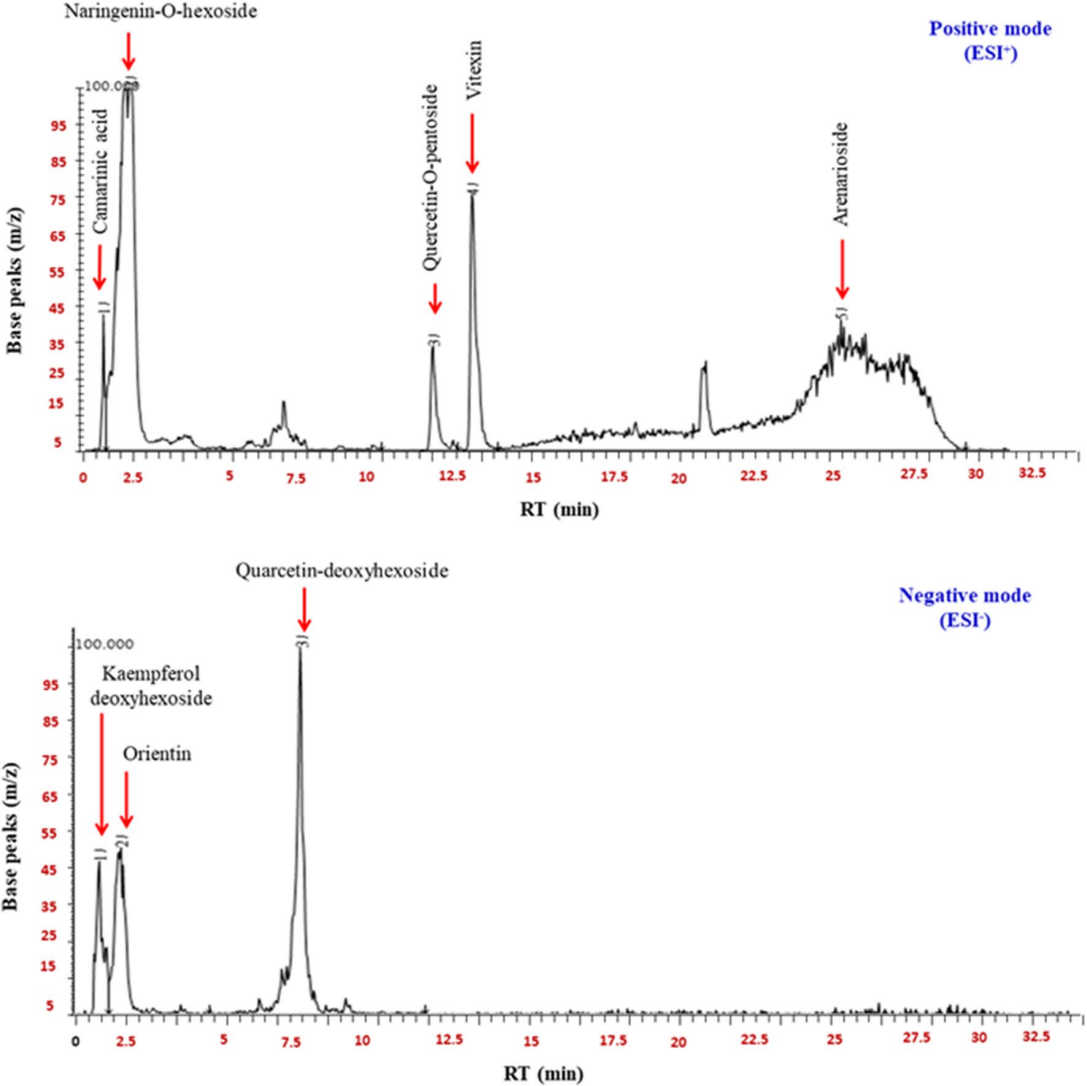
LC–MS chromatograms of CS-PAE.

The identification of CS-PAE ingredients depended on four representative indices, GC–MS, phytochemical composition estimation, phenolic profile assessment via HPLC, and chemical profiling using LC–MS. The GC–MS analysis recognized some bioactive compounds (Fig. 1), and their biological activities are informed in Table S1. The second direction of CS-PAE component identification was phytochemical estimation by determining TPC, TFC, and total carbohydrate, lipid, and protein content (Table 1). Data in Table 2 exhibited the concentration of different studied phenolic and flavonoid compounds. The CS-PAE contains a higher concentration of GA, protocatechuic acid (PCA), and apigenin-7-glucoside (646.951, 140.306, and 44.378 µg/g, respectively), and the HPLC chromatograms were shown in Fig. 2. Further, the LC–MS of CS-PAE disclosed the existence of 25 compounds. In contrast, ten compounds were recognized in positive and negative ionization mods belonging to distinct classes of secondary metabolites (Table 3, Fig. 3).

**Table 1:** Phytochemical composition of CS-PAE.

	Phytochemical constituent
TPC	9.07 ± 0.21 µg GA equivalent/mg CS-PAE
TFC	6.68 ± 0.09 µg QUR equivalent/mg CS-PAE
Total carbohydrate content	43.58 ± 0.27 µg glucose equivalent/mg CS-PAE
Total lipids content	53.65 ± 0.43 µg palmitic acid equivalent/mg CS-PAE
Total protein content	32.12 ± 0.59 µg bovine albumin equivalent/mg CS-PAE

Values represent the mean ± SE of three samples using the SPSS program (Version 25, ANOVA one-way test).

**Table 2:** Phenolic profile of CS-PAE.

Compound	RT	Concentration (µg/g)
Gallic acid	3.9	646.951 ± 0.02
Protocatechuic acid	7.7	140.306 ± 0.01
*p*-hydroxybenzoic	12.1	4.669 ± 0.01
Catechin	15.2	1.611 ± 0.03
Caffeic acid	17.2	3.005 ± 0.01
Syringic acid	19.3	3.299 ± 0.02
Vanillic acid	21.2	2.735 ± 0.01
Ferulic acid	28.9	4.049 ± 0.01
Sinapic acid	30.7	0.686 ± 0.01
Rutin	34.5	4.785 ± 0.01
*p*-coumaric acid	35	6.033 ± 0.02
Apigenin-7-glucoside	38.2	44.378 ± 0.02
Rosmarinic	39.2	0.565 ± 0.01
Cinnamic	46.9	3.245 ± 0.01
Quercetin	49.5	0.832 ± 0.01
Apigenin	55.2	0.282 ± 0.02
Kaempferol	55.9	0.377 ± 0.01
Chrysin	59	0.375 ± 0.01

Values represent the mean ± SE of three samples using the SPSS program (Version 25, ANOVA one-way test).

**Table 3:** Compounds detected in LC–MS analysis of CS-PAE.

	Compound name	Chemical class	Molecular formula	Molecular weight
1	Naringenin-O-hexoside	Flavonols	C_12_H_22_O_10_	435.30
2	Quercetin-O-pentoside	Phenolic	C_20_H_18_O_11_	435.30
3	Arenarioside	Phenyl ethanoid glycosides	C_34_H_44_O_19_	757
4	Kaempferol deoxyhexoside	Phenolic		431.35
5	Vitexin	Phenolic	C_21_H_19_O_10_	431.35
6	Dihydroxy-dimethoxy flavone hexoside II	Flavonoids	C_23_H_24_O_11_	475
7	Pectolinarin	Flavonoids	C_29_H_34_O_15_	620
8	Camarinic acid	Ursane-type triterpene	C_32_H_48_O_6_	529
9	Orientin	Phenolic	C_21_H_19_O_11_	447.25
10	Quarcetin-deoxyhexoside	Phenolic	C_21_H_20_O_12_	447.25

### Antioxidant activity of CS-PAE

**Figure 4:**
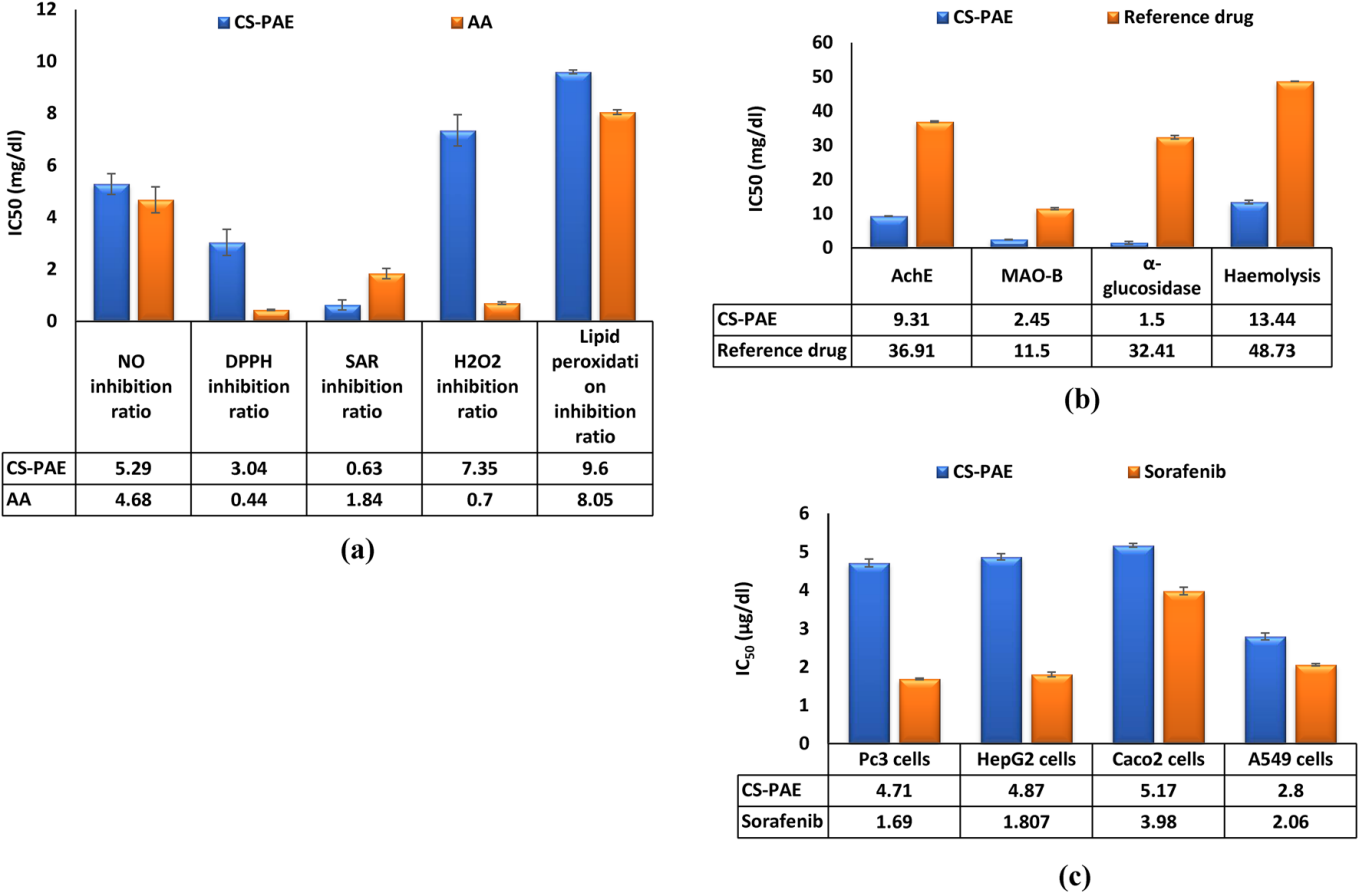
IC_50_ of CS-PAE and studied reference drugs of some analyzed parameters, **(a)** IC_50_ of CS-PAE and AA in studied antioxidant parameters, **(b)** IC_50_ of CS-PAE and reference drugs in studied neuro-inflammatory, antidiabetic, and anti-inflammatory parameters, **(c)** IC_50_ of CS-PAE and sorafenib in studied tumor cells.

Phenolic compounds in CS-PAE confer a remarkable antioxidant power linked to the existence of hydroxyl (OH) groups and the double-bond conjugation in most phenols. At the same time, polyphenols in CS-PAE could scavenge reactive oxygen species (ROS) and create more stable phenoxyl radicals, which reflects in the CS-PAE's powerful antioxidant behavior^50^. The antioxidant activity of CS-PAE was evaluated and compared with AA by several assays of TAC, FRP, NO, DPPH, SAR, H_2_O_2,_ and lipid peroxidation (Tables 4, 5). Additionally, CS-PAE IC_50_ was recorded in Fig. 4a.

**Table 4:** TAC (mg/ml), FRP (mg/ml) and NO and DPPH scavenging inhibition % of CS-PAE and AA.

Concentrations (mg/ml)	TAC (mg/ml)		FRP (mg/ml)		NO scavenging inhibition %		DPPH scavenging inhibition %	
	CS-PAE	AA	CS-PAE	AA	CS-PAE	AA	CS-PAE	AA
0.8	0.33 ± 0.02	0.95 ± 0.01	11 ± 0.01	16.9 ± 0.03	2.2 ± 0.11	24.62 ± 0.2	27.8 ± 0.04	67.81 ± 0.04
1	0.37 ± 0.01	1.5 ± 0.01	11 ± 0.01	20.2 ± 0.09	3.35 ± 0.18	33.63 ± 0.06	35.46 ± 0.1	68.91 ± 0.1
2	1.65 ± 0.22	2.26 ± 0.02	23 ± 0.01	25.9 ± 0.21	26.32 ± 0.02	42.47 ± 0.03	49.39 ± 0.07	70.18 ± 0.02
4	4.76 ± 0.2	5.89 ± 0.03	33 ± 0.02	41.4 ± 0.11	52.41 ± 0.04	52.7 ± 0.05	62.79 ± 0.02	71.31 ± 0.02
6	6.65 ± 0.13	8.5 ± 0.19	37 ± 0.01	60.8 ± 0.36	63.14 ± 0.07	60.08 ± 0.03	72.99 ± 0.04	72.45 ± 0.11
8	7.03 ± 0.33	10.78 ± 0.23	45 ± 0.02	69.8 ± 0.05	73.43 ± 0.05	63.84 ± 0.04	78.56 ± 0.06	73.14 ± 0.02
10	9.63 ± 0.49	14.02 ± 0.09	63 ± 0.03	71.1 ± 0.15	82.99 ± 0.08	68.44 ± 0.03	83.74 ± 0.07	73.71 ± 0.01

Values represent the mean ± SE of three samples using the SPSS program (Version 25, ANOVA one-way test).

**Table 5:** SAR, H_2_O_2_ and lipid peroxidation scavenging inhibition % of CS-PAE and AA.

Concentrations (mg/ml)	SAR scavenging inhibition %		H_2_O_2_ scavenging inhibition %		Lipid peroxidation scavenging inhibition %	
	CS-PAE	AA	CS-PAE	AA	CS-PAE	AA
0.8	69.97 ± 0.05	50.54 ± 0.03	43.99 ± 0.06	74.09 ± 0.09	7.22 ± 0.05	9.23 ± 0.03
1	72.78 ± 0.07	55.31 ± 0.06	45.33 ± 0.07	75.58 ± 0.04	8.06 ± 0.07	10.89 ± 0.06
2	79.43 ± 0.09	65.1 ± 0.07	47.17 ± 0.07	80.32 ± 0.03	16.66 ± 0.09	15.05 ± 0.07
4	85.23 ± 0.02	76.85 ± 0.06	48.67 ± 0.02	83.06 ± 0.03	19.05 ± 0.02	29.6 ± 0.06
6	87.76 ± 0.03	84.94 ± 0.08	49.6 ± 0.09	85.92 ± 0.04	35.23 ± 0.03	44.2 ± 0.08
8	90.38 ± 0.09	89.66 ± 0.09	50.34 ± 0.07	86.93 ± 0.03	43.8 ± 0.09	50.09 ± 0.09
10	92.89 ± 0.04	95.76 ± 0.01	51.17 ± 0.06	88.06 ± 0.07	50.47 ± 0.01	57.02 ± 0.04

Values represent the mean ± SE of three samples using the SPSS program (Version 25, ANOVA one-way test).

It was disclosed that GA, rutin (RUT), and catechin (CAT) in our examined CS-PAE composition have valuable, interesting antioxidant activity. GA is characterized by its free radical-neutralizing biological functions, and at the same way RUT has the aptitude to scavenge the OH and donate the hydrogen. CAT is vital in protecting against generated ROS due to the existence of the catechol group in its phenolic B ring and OH and galloyl groups in its chemical structure. Further, the present study showed the elevation of FRP by increasing CS-PAE concentration which may be interpreted due to the electron-donating capability of phenolic content in CS-PAE^51^. In addition, GC–MS of CS-PAE revealed some components that possessed antioxidant power (Table S1). Our results were matched with Hsouna et al.^52^, who revealed that the ethyl acetate fraction of CS exhibited more OH radicals' inhibition than the antioxidant reference.

### The anti-inflammatory, antidiabetic, and neurological activity of CS-PAE

The current data exhibited the ability of CS-PAE to prevent the RBCs hemolysis and the inflammation process (Table 6). Such is perhaps ascribed to the contribution of RUT as one of the phenolic compounds in the examined extract, which obstructs some key inhibitory enzyme activity involved in the inflammation process, such as myeloperoxidase found in infiltrated neutrophils^53^. Recent studies exhibited that RUT declined the caspase-3 cleavage and inhibited the release of high mobility group box 1, a mediator of the severe vascular inflammatory disorder^54^. At the same time, it was proved that kaempferol (KAEM), another phenolic compound in CS-PAE, is vital in decreasing inflammation via inhibition of NO and lipopolysaccharides creation^55^. Additionally, the anti-inflammatory activity may be related to detecting some compounds in our examined extract (Table S1).

**Table 6:** Observed RBCs hemolysis, α-glucosidase enzyme, and AchE and MAO-B activities inhibitory % of CS-PAE different concentrations compared to reference drug.

Concentrations (mg/ml)	RBCs hemolysis inhibitory %		α-glucosidase enzyme inhibitory %		AchE activitiy inhibitory %		MAO-B activitiy inhibitory %	
	CS-PAE	Diclofenac potassium	CS-PAE	Metformin	CS-PAE	Donepezil	CS-PAE	Donepezil
0.8	10.0 ± 0.03	25.54 ± 0.02	20.06 ± 0.03	60.93 ± 0.04	7.5 ± 0.02	2.36 ± 0.08	96.62 ± 0.1	77.36 ± 0.05
1	23.3 ± 0.08	31.49 ± 0.02	40.75 ± 0.1	63.34 ± 0.02	10.92 ± 0.08	3.53 ± 0.04	96.93 ± 0.02	88.29 ± 0.02
2	34.0 ± 0.09	45.26 ± 0.03	50.89 ± 0.03	65.4 ± 0.04	22.94 ± 0.07	5.83 ± 0.1	97.27 ± 0.09	88.7 ± 0.06
4	50.2 ± 0.01	62.03 ± 0.05	70.44 ± 0.1	80.19 ± 0.03	33.58 ± 0.04	9.46 ± 0.02	97.74 ± 0.1	88.94 ± 0.02
6	70.65 ± 0.04	74.4 ± 0.01	74.53 ± 0.1	85.3 ± 0.02	38.07 ± 0.09	11.06 ± 0.06	98.6 ± 0.1	89.56 ± 0.05
8	76.44 ± 0.03	92.9 ± 0.1	83.02 ± 0.03	92.51 ± 0.02	43.64 ± 0.03	12.8 ± 0.01	99.56 ± 0.05	89.0 ± 0.04
10	81.36 ± 0.03	99.0 ± 0.03	90.49 ± 0.03	98.0 ± 0.1	49.47 ± 0.03	14.63 ± 0.02	99.83 ± 0.02	89.86 ± 0.08

Values represent the mean ± SE of three samples using the SPSS program (Version 25, ANOVA one-way test).

The antioxidant and anti-inflammatory activity of CS-PAE positively impact the development of chronic diseases such as diabetes mellitus and neurodegenerative disorders. The antihyperglycemic effect of CS-PAE was evaluated and compared with metformin by α-glucosidase inhibition activity assay (Table 6), and the IC_50_ was recorded in Fig. 4b. CS-PAE can prevent α-glucosidase enzyme activity, which works upon terminal α-1,4-glycosidic linkage of starch, glycogen, and disaccharides and catabolizes them in the gastrointestinal tract into glucose^56^. Inhibition of that enzyme is one of the key factors responsible for adjusting blood glucose levels due to the reduction of intestinal carbohydrate uptake. The α-glucosidase inhibitory effects of CS-PAE may be accredited to its richness in varied phytochemicals, including phenolics and flavonoids like QUR, GA, RUT, and KAEM. In this way, the assumptions of Custódio et al.^57^ were similar to our results, where the in vitro inhibitory activity of CS-PAE on the α-glucosidase enzyme was reported.

Further, a previous study demonstrated that GA could control phosphatidylinositol 3-kinase/protein kinase B and adenosine monophosphate‐activated protein kinase signaling mechanisms, thus having a role in hyperglycemia recovery^58^. Moreover, the presence of phytochemical components, PCA and vanillic acid, in CS-PAE were involved in the process of α-glucosidase enzyme inhibition due to many OH groups found in the chemical structure of those compounds, which enable them to chelate the α-glucosidase enzyme via modification in its structure, so the enzyme wasted its biological activity^59^. Another hypothesis of CS-PAE α-glucosidase inhibitory influence was credited to the existence of some components (Table S1).

Further, the in vitro study results of CS-PAE displayed its potency to inhibit the activity of AchE (Table 6) due to its higher content in phenolic compounds, chiefly flavonoids, that have been recognized for their AchE-obstructing activity^60^. Our results exposed the presence of GA and PCA in CS-PAE, which are distinguished by the presence of phenolic OH groups and can scavenge ROS, minimize inflammation in neurodegenerative diseases, and modulate the neurotransmitter acetylcholine concentration in the hippocampus and cortex of the brain^61^. Our findings concurred with Sekeroglu et al.^62^, who stated that CS's high antioxidant activity could inhibit AchE activity. Also, Uysal et al.^63^ showed that aqueous extract of CS leaves had AchE inhibitory activity. It was also observed that IC_50_ in the case of CS-PAE (9.31 mg/ml) was lower than donepezil which was (36.91 mg/ml) (Fig. 4b).

Moreover, MAO-B inhibitors are considered another target to fight some neurodegenerative diseases. Results in the present study exhibited that CS-PAE had a powerful inhibitory % against MAO-B (Table 6), and that may be explained due to the presence of QUR, KAEM, and apigenin, even in their low concentration. Until now, there isn't a previous work link between CS or its extracts and MAO-B, but previous works on other herbal extracts attributed the repressing activity to phenolic and flavonoid chemicals^64^. Our data was shown that CS-PAE has various components that serve a neuronal role (Table S1).

### Antiproliferative effect of CS-PAE

**Figure 5:**
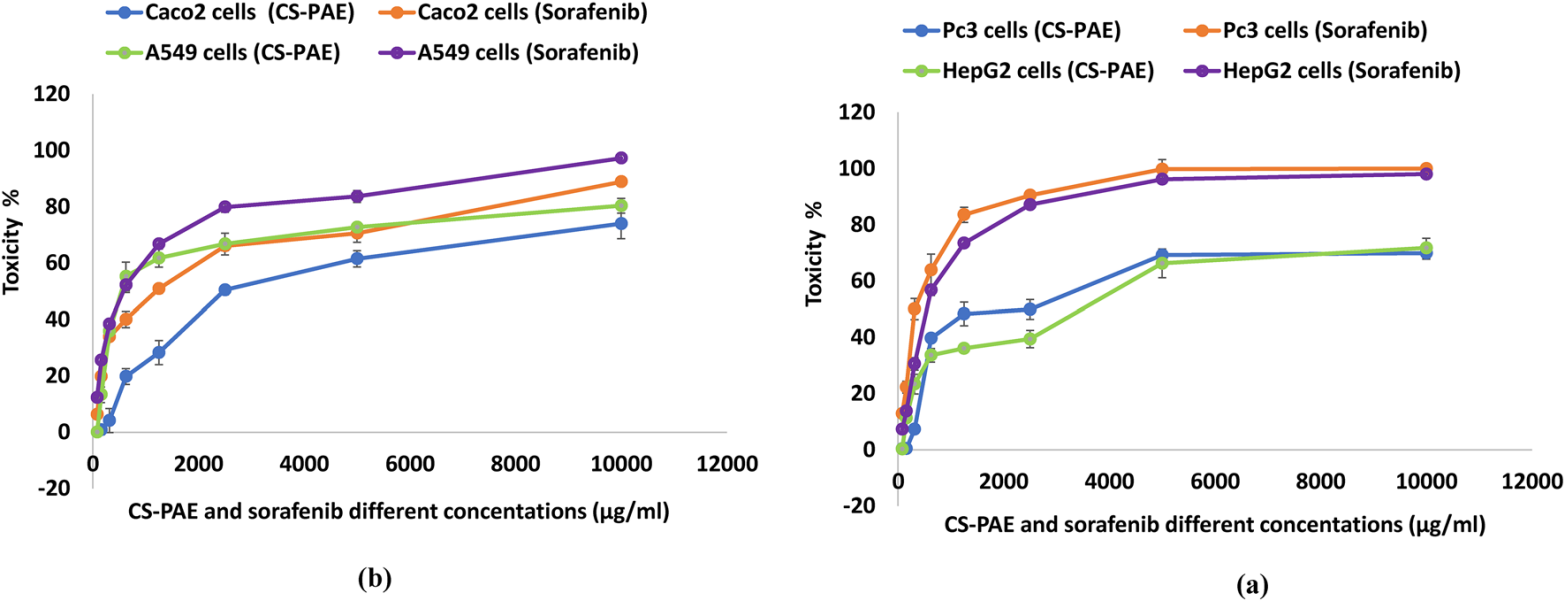
Effect of CS-PAE and sorafenib different concentrations on tumor cell lines, **(a)** prostate cancer Pc3 and liver cancer HepG2 and **(b)** colon cancer Caco2 and lung cancer A549.

The study displayed the close relation between the anticancer effects of CS-PAE and its anti-inflammatory and antioxidant activity by various supposed theories on different examined tumor cells, Pc3, HepG2, Caco2, and A549. The data were compared with the reference drug sorafenib and summarized in Fig. 5a,b, and the IC_50_ was mentioned in Fig. 4c. Analysis of CS-PAE by GC–MS showed eleven components with anticancer activity (Table S1).

Also, HPLC results exhibited different components had antineoplastic roles. Firstly, GA, the most abundant polyphenolic compound in the CS-PAE, could obstruct IκB kinase-ß/NF-ĸB signal stimulation; consequently, the expression of inflammatory mediators could be declined^65^. Also, GA, QUR, and KAEM perform their anticancer influence via cell cycle arrest and lessen the ratio of cells in the G0/G1 phase^66^. Previous studies by Stavrou et al.^67^ explained that both GA and QUR were cytotoxic agents via their intermediate polarity and had a vital role as antiproliferative. Moreover, RUT, a phenolic compound in the extract under study, modulates the action of cytochrome P450 and phases II enzymes. These enzymes function critically in cellular defense against producing free radicals and toxic metabolites during the cancer cell cycle^68^.

### Antiviral activity of CS-PAE

**Figure 6:**
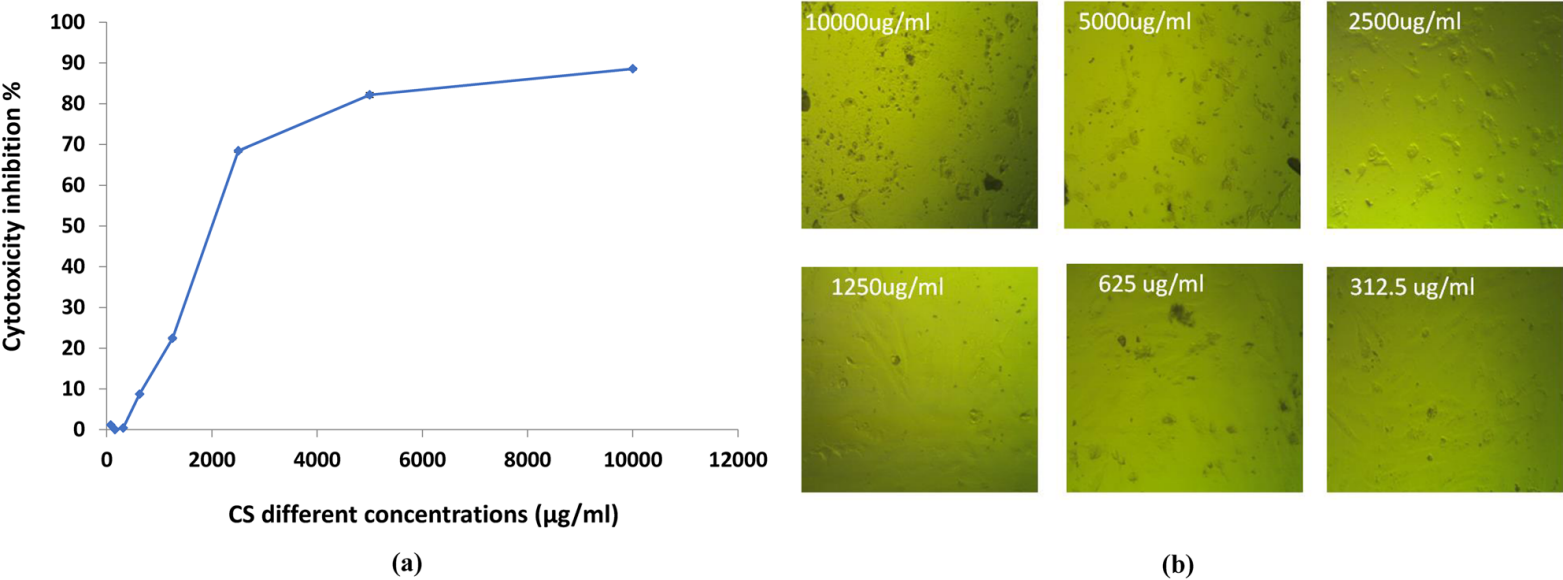
Effect of CS-PAE different concentrations on VERO cell cytotoxicity, **(a)** cytotoxicity inhibition % and **(b)** microscopical images of CS-PAE different concentrations effect on VERO cells.

**Figure 7:**
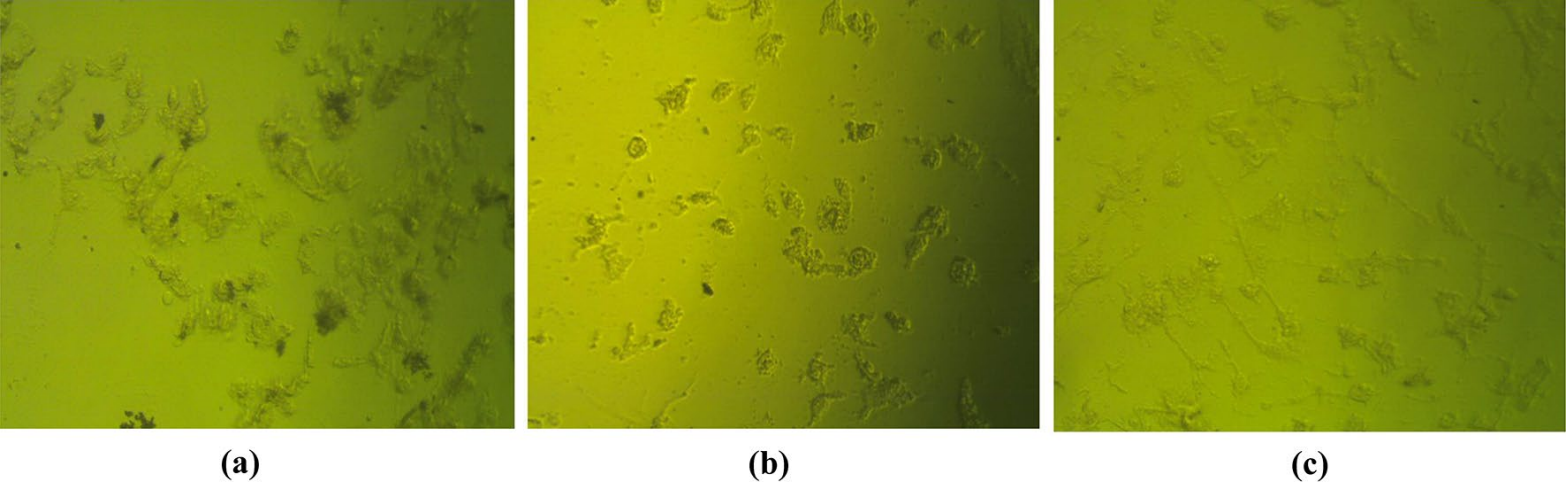
Microscopical images of the effect of studied viruses on VERO cells, **(a)** effect of HAV on VERO cells, **(b)** effect of CoxB4 on VERO cells, and **(c)** effect of HSV1 on VERO cells.

**Figure 8:**
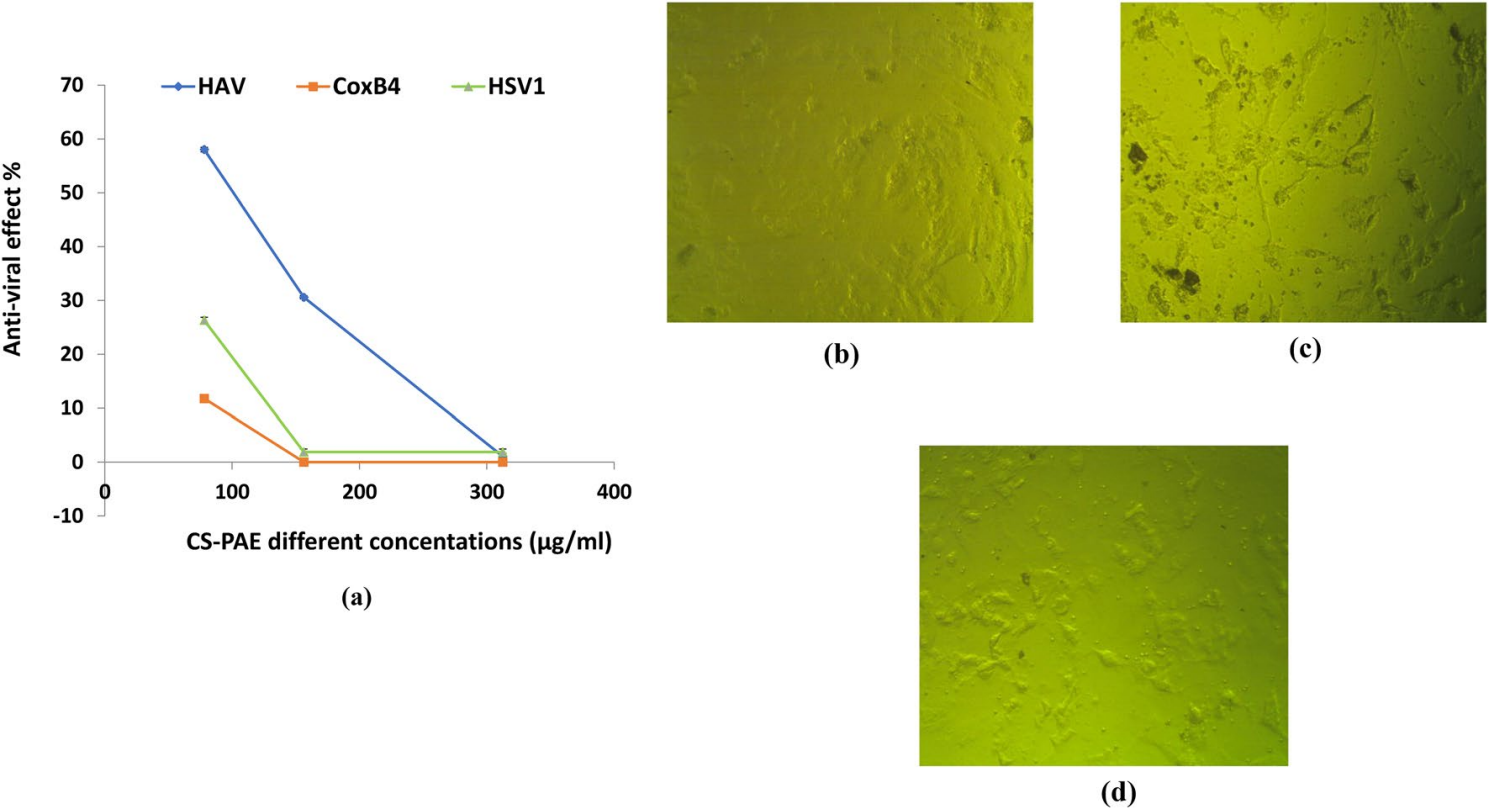
Antiviral effect of MNTC different concentrations of CS-PAE on infected VERO cells with studied viruses, **(a)** antiviral effect % of MNTC different concentrations of CS-PAE on infected VERO cells with studied viruses, **(b)** microscopical image of the effect of CS-PAE at concentration 312.5 µg/ml on infected VERO cells with HAV, **(c)** microscopical image of the effect of CS-PAE at concentration 312.5 µg/ml on infected VERO cells with CoxB4, **(d)** microscopical image of the effect of CS-PAE at concentration 312.5 µg/ml on infected VERO cells with HSV1.

The cytotoxicity test was conducted versus VERO cells to ascertain the safety of CS-PAE. VERO cells were an excellent in vitro research model because of their toxicity susceptibility. The results showed that CS-PAE had cytotoxicity on VERO cells with CC_50_ less than 312.5 μg/ml (Fig. 6). Microscopically examining HAV, CoxB4, and HSV1 revealed an irregular outline, cytoplasmic projections, and intense cytoplasmic vacuolization. In addition, the nuclear membrane started to be disintegrated. The cytoplasm appeared as a mottled, and there was a diffusion of mass throughout the cytosol with compact lysosomes and myelin figures (Fig. 7a–c). The non-toxic protective influence of CS-PAE at different concentrations (78.12, 156.25, and 312.5) µg/ml against HAV, CoxB4, and HSV1 was studied (Fig. 8a). Microscopical examination of tested CS-PAE at a concentration of 312.5 µg/ml was implemented and illustrated in (Fig. 8b–d).

The antiviral activity of CS-PAE against HAV may be ascribed to the phenolic compounds' existence, which had a potent antioxidant power to fight ROS produced by the virus^69^. According to previous studies, phenols interfere with the virus's adsorption, entry, and binding^70^. They also hinder the synthesis of protein complexes, deoxyribonucleic acid, and ribonucleic acid polymerases^71^. On the other hand, the antiviral efficacy of CS-PAE against HSV-1 was lower than HAV, and this could be clarified due to the minimum concentration of ferulic acid, QUR, and CAT, which are mainly responsible for fighting such type of virus via the inactivation process^72^. The results reported that CS-PAE has very little influence against CoxB4. Additionally, there were multiple components illustrated in (Table S1) owned antiviral efficacy.

### Antimicrobial activity of CS-PAE

The results in Table 7 and Fig. S1 disclosed that gentamycin had a significant influence against pathogenic bacteria (*Bacillus subtilis*, *Staphylococcus aureus*, *Klebsiella pneumonia,* and *Escherichia coli*) in comparison to CS-PAE, which had an effect only against *Bacillus subtilis* and *Klebsiella pneumonia*. The antibacterial ability of CS-PAE may be related to the impact of glycosylation and the methylation processes of CS-PAE phenolic components to interact with bacterial membrane structure^73^. Further, the in vitro antifungal of CS-PAE was performed against *Candida albicans* and *Aspergillus flavus,* and its efficacy was compared with standard amphotericin. The values indicate that amphotericin may be a good inhibitor of the selected fungus, while CS-PAE wasn't effective. Our examined extract found various elements and revealed antimicrobial and antifungal activity (Table S1).

**Table 7:** Antimicrobial activity (µg/ml) of CS-PAE.

Studied strains		CS-PAE	Gentamycin	Amphotericin
Gram-positive bacteria strains	*Bacillus subtilis*	18.62 ± 0.06***	26.10 ± 0.06	No activity
	*Staphylococcus aureus*	No activity	18 ± 0.17	No activity
Gram-negative bacteria strains	*Klebsiella pneumoniae*	13.62 ± 0.6***	22.10 ± 0.06	No activity
	*Escherichia coli*	No activity	24 ± 0.37	No activity
Fungus strains	*Candida albicans*	No activity	No activity	19 ± 0.25
	*Aspergillus flavus*	No activity	No activity	15 ± 0.19

Values represent the mean ± SE of three samples using the SPSS program (Version 25, ANOVA one-way test). Significance: *P < 0.05; **P < 0.01; ***P < 0.001 comparing to reference drug.

### Molecular docking of CS-PAE on the studied biomarkers

The existing research aims to study the uppermost CS-PAE components that interfere with target proteins that have a known role in mediating oxidative stress and inflammatory pathways. Molecular docking of the top hit compounds to validate the interaction pattern to the crucial target protein Keap-1/ Nrf2 was illustrated in Table 8. They were ordered according to their docking affinity scores: quercetin-O-pentoside > RUT > naringenin-O-hexoside > apigenin-7-glucoside > *p*-coumaric acid. It was noticed that the docking scores of quercetin-O-pentoside, RUT, naringenin-O-hexoside, apigenin-7-glucoside, and *p*-coumaric acid were − 8.50, − 7.66, − 7.06, − 6.65 and − 6.58 kcal/mol, respectively. The docking score of all studied compounds was higher than the co-crystalized ligand (RA839), which was − 6.12 kcal/mol.

**Table 8:** The top hit compounds in the CS-PAE-target network against the Keap-1/ Nrf2 target protein.

Studied compounds and co-crystallized ligand	RMSD value (Å)	Binding affinity score (Kcal/mol)	Interactions	
			Number of Hydrogen bonds	Number of Pi-interaction
Co-crystallized ligand (RA839)	1.21	− 6.12	4	10
Quercetin-O-pentoside	1.42	− 8.50	3	5
Rutin	1.81	− 7.66	4	2
Naringenin-O-hexoside	1.12	− 7.06	4	2
Apigenin-7-glucoside	1.56	− 6.65	4	3
*p*-coumaric acid	1.85	− 6.58	7	-

The docked pose of RA839 against Keap-1/Nrf2 was represented in Fig. S2a–c. It was distinguished that it fitted well into the active site via forming ten Pi-Pi, and Pi-Alkyl interactions with Tyr334, Tyr572, Phe577, Ala556, and Arg415. Additionally, RA839 interacts with Ser363, Ser602, Ser508, and Arg483 with four hydrogen bonds with a distance of 2.62, 2.01, 2.16, and 2.12 Å. Moreover, RA839 is bound to the core protein through attractive interactions with the amino acid residues Arg415 and Arg483.

Comparing the 2D and 3D interaction diagram of quercetin-O-pentoside (Fig. S3a–c) to that of RA839, it demonstrated the formation of five Pi-Alkyl interactions with Arg415 and Ala556. In addition, it interacted with Asn414, Asn382, and Arg483 by three hydrogen bonds with a distance of 2.02, 1.84, and 1.98 Å. However, RUT formed two Pi-Alkyl and Pi-Pi interactions with Tyr525 and Tyr334 and was involved in three hydrogen bonds (Asn387, Asn414, and Arg415) with a distance of 3.04, 2.12, 2.41, and 3.04 Å (Fig. S4a–c). Further, naringenin-O-hexoside formed two Pi-Alkyl and Pi-cation interactions with Arg415 and Ala556. It was incorporated in four hydrogen bonds (Arg415, Ser602, Arg483, and Ser508) with bond lengths of 5.02, 2.40, 2.06, and 2.13 Å (Fig. S5a–c). In addition, apigenin-7-glucoside was engaged in three Pi-Alkyl and Pi–Pi interactions with Ala556, Arg415, and Tyr334. It also displayed four hydrogen bonds with Ser555, Arg483, and Ser363 with bond lengths of 2.87, 5.26, 2.78, and 2.03 Å (Fig. S6a–c). From Fig. S7a–c, it was postulated that *p*-coumaric acid was binding with Ser602, Asn382, Asn414, Arg415, Arg483, and Ser508 with seven hydrogen bonds with bonds lengths 1.85, 2.30, 2.22, 4.90, 1.88, 2.53 and 1.89 Å.

Further, as depicted in Table 9, the tested compounds interacted with disparate binding affinities in the active site of NF-ĸB protein and were ranked according to their binding affinity scores as follows: apigenin-7-glucoside > RUT > *p*-coumaric acid > GA. It was observed that apigenin-7-glucoside and RUT explored higher docking scores of − 8.43 and − 7.95 kcal/mol, respectively, compared to co-crystalized ligand (benzoxepin) (− 6.78 kcal/mol). The representative combined site of benzoxepin against NF-ĸB was illustrated in Fig. S8a–c. Herein, it was found that benzoxepin formed twelve Pi-Alkyl, Pi-sigma, and Pi-sulfur interactions with Met471, Lys431, Lys535, Leu524, Arg 410, and Val416, additionally, it interacted with Leu474 and Glu472 with two hydrogen bonds with a distance of 2.54 and 1.87 Å.

**Table 9:** The top hit compounds in the CS-PAE-target network against the NF-ĸB target protein.

Studied compounds and co-crystallized ligand	RMSD value (Å)	Binding affinity score (Kcal/mol)	Interactions	
			Number of Hydrogen bonds	Number of Pi-interaction
Co-crystallized ligand (Benzoxepin)	1.32	− 6.78	2	12
Apigenin-7-glucoside	1.45	− 8.43	4	12
Rutin	1.59	− 7.95	9	11
*p*-coumaric acid	1.85	− 6.35	4	–
Gallic acid	1.72	− 5.86	3	3

The studied compound, apigenin-7-glucoside, versus NF-ĸB, predicted twelve Pi-Alkyl, Pi-sulfur, and Pi-sigma interactions with Cys535, Mrt471, Cys446, Lys431, Ile469, and Val416. Moreover, it bound with Gln481, Leu474, Glu472, and Cys446 by four hydrogen bonds with bond lengths of 1.97, 2.29, 2.474, and 2.62 Å (Fig. S9a–c). Analogously, RUT formed eleven Pi-sulfur and Pi-Alkyl interactions with Met471, Arg410, Leu524, Val416, Cys535, and Lys431. Additionally, it interacted with Glu442, Met471, Leu474, Gln481, Asp536, Ser412, and Gly411 by nine hydrogen bonds with a distance of 2.40, 2.44, 2.43, 2.68, 2.19, 2.57, 1.71, 1.83 and 2.07 Å (Fig. S10a–c). Furthermore, the binding mode of *p*-coumaric acid displayed an affinity binding energy of − 6.35 kcal/mol against the NF-ĸB target site. *p*-coumaric acid bound with Arg410, Leu474, Lys519, and Asp536 with four hydrogen bonds with bonds lengths of 2.12, 2.38, 2.05, and 2.77 Å (Fig. S11a–c). The binding mode of GA displayed an energy binding of − 5.86 kcal/mol against the NF-ĸB target site. From Fig. S12a–c, GA predicted three Pi-Alkyl interactions with Val416, leu524, and Arg410. Also, it incorporated with Leu474 and Arg410 by three hydrogen bonds with bond lengths of 2.38, 1.90, and 2.42 Å.

Previous results showed that a high ligand posture docking score denotes a more positive orientation; the most popular Nrf2 activator had a docking average of less than − 6.12. Also, the recognized NF-ĸB inhibitors had an average docking energy score of fewer than − 6.78 (the minimum value, the highest score) relative to the reference candidate. Quercetin-O-pentoside exhibited a better binding mode with Nrf2, allowing it to endorse cell apoptosis and reduce the metastasis process of neoplasms^74^. In the same way, apigenin-7-glucoside displayed a potent interaction within Nrf2 and NF-ĸB leading to cell cycle arrest and anti-invasive activity^75^. The results indicated that the selected compounds with high binding affinity with Nrf2 could directly inhibit NF-ĸB protein–protein interaction. So, these compounds may have direct or indirect therapeutic effects on Keap1, encourage Nrf2 nuclear translocation, and inhibit NF-ĸB. Therefore, they can initiate the transcriptional steps to improve the control of the antioxidant defense system and inflammatory response.

## Conclusions

Our research disclosed that the signaling pathways adjustment accompanied by numerous pathologic settings by focusing on their crucial protein components could act as molecular targets for therapy and-/or protection of metabolic illnesses. *Ceratonia siliqua* pulp aqueous extract (CS-PAE) contained a high percentage of gallic acid, protocatechuic acid, apigenin-7-glucoside, carbohydrates, lipids, and proteins. So, CS-PAE owned antioxidant and anti-inflammatory activity. Further, it possessed anticancer activity against prostate cancer Pc3, liver cancer HepG2, colon cancer Caco2, and lung cancer A549. Also, the studied extract had an antiviral influence against the hepatitis A virus, but there are some limitations in Coxsackievirus B4 and herpes simplex virus 1. The CS-PAE also exposed antimicrobial activity against some pathogenic Gram-positive and negative bacteria such as *Bacillus subtilis*, *Staphylococcus aureus*, *Klebsiella pneumonia,* and *Escherichia coli*; however, there is a restriction to its antifungal outcome against *Candida albicans* and *Aspergillus flavus*.

After screening all CS-PAE candidates against Keap-1/ Nrf2 and NF-ĸB targeted receptors, they show a high affinity of apigenin-7-glucoside, rutin, and *p*-coumaric acid toward Keap-1/Nrf2 and NF-ĸB. However, quercetin-O-pentoside and naringenin-O-hexoside displayed a potent affinity toward Keap-1/ Nrf2 and gallic acid-induced good affinity toward NF-ĸB, which conveys an image of the predictable activity of CS-PAE as an exceptional anti-inflammatory.

Considering the results of this research, we think that CS-PAE has yielded a beneficial consequence and can be used as a helpful therapeutic agent for several chronic diseases such as diabetes mellitus, neurodegenerative diseases, oncogenic and some viral and microbial disorders. However, this requires further in vivo studies to grasp its various mechanisms of action.

## Supplementary Information

Supplementary Information.

## Data Availability

The data supporting this study's findings are available from the corresponding author upon reasonable request.
