# Supplementary material for: Pharmacological bioactivity of Ceratonia siliqua pulp extract: in vitro screening and molecular docking analysis, implication of Keap-1/Nrf2/NF-ĸB pathway
Source: Sci Rep. 2023 Jul 27;13:12209. doi: 10.1038/s41598-023-39034-4 (PMC10374561; doi:10.1038/s41598-023-39034-4)
Supplement: Supplementary file 1 — Supplementary Information. [file 41598_2023_39034_MOESM1_ESM.docx]

**Supplementary Data**

**Table S1. Compounds detected in GC-MS analysis of CS-PAE**

|  | RT | Area % | Compound name | Molecular formula | Molecular weight | MF | Compound biological activity |
| --- | --- | --- | --- | --- | --- | --- | --- |
| 1. | 4.56 | 0.66 | 17-Octadecynoic acid | C_18_H_32_O_2_ | 280 | 535 | Anti-hyperlipidemic and antiatherosclerosis activity |
|  |  |  | 9,12,15-Octadecatrienoic acid, 2-(acetyloxy)-1 (acetyloxy) methyl-ethyl ester-(Z,Z,Z) | C_25_H_40_O_6_ | 436 | 537 | Anti-inflammatory ^1^ , anti-hypercholesterolemic, anticancer ^2^, anti-coronary, and hepatoprotective activity |
| 2. | 4.91 | 0.60 | Melezitose | C_18_H_32_O_16_ | 504 | 636 | Anticancer ^2^ and anti-infection activity ^2^ |
| 3. | 5.26 | 1.10 | 4-Cyclopentene-1,3-dione | C_5_H_4_O_2_ | 96 | 826 | Not known |
| 4. | 5.39 | 1.40 | Cyclopent-2-En-1,4-dione | C_5_H_4_O_2_ | 96 | 944 | Not known |
| 5. | 5.61 | 0.85 | Xylose | C_5_H_10_O_5_ | 150 | 653 | Not known |
| 6. | 5.74 | 4.72 | 2-Cyclopenten-1-one-2-hydroxy | C_5_H_6_O_2_ | 98 | 994 | Inducer of Heat Shock Protein 70 with antiviral activity ^3^ |
|  |  |  | 2-Hdroxycyclopent-2-EN-1-One | C_5_H_6_O_2_ | 98 | 983 | Anticancer activity ^4^ |
| 7. | 6.73 | 1.03 | 2(5H)-Furanone | C_4_H_4_O_2_ | 84 | 873 | Antimicrobial activity ^5^ |
| 8. | 7.58 | 0.46 | 3,5-dichloropyridine-1-oxide | C_5_H_3_C_l2_NO | 163 | 628 | Not known |
| 9. | 8.0 | 1.24 | Glycerin | C_3_H_8_O_3_ | 92 | 888 | Not known |
| 10. | 8.54 | 12.13 | Formic acid-2-propenyl ester | C_4_H_6_O_2_ | 86 | 908 | Antifungal activity ^6^ |
| 11. | 8.82 | 0.91 | Melezitose | C_18_H_32_O_16_ | 504 | 628 | Anticancer and anti-infection activity |
| 12. | 9.19 | 1.23 | 11-Aminoundecanoic acid | C_11_H_23_NO_2_ | 201 | 595 | Not known |
| 13. | 9.42 | 0.95 | 2H-Pyran-2-methanol-tetrahydro | C_6_H_12_O_2_ | 116 | 707 | Not known |
| 14. | 9.72 | 0.98 | Fumaric acid-3-methylbut-3-enyl undecyl ester | C_20_H_34_O_4_ | 338 | 734 | Not known |
| 15. | 10.10 | 4.26 | 4H-Pyran-4-one-2,3-dihydro-3,5 dihydroxy-6-methyl | C_6_H_8_O_4_ | 144 | 899 | Catechol-O-methyl-transferase inhibitor, methyl guanidine inhibitor, and methyl donor |
|  |  |  | 2,3-Dihydro-3,5-dihydroxy-6-methyl-4H-Pyran-4-One | C_6_H_8_O_4_ | 144 | 910 | Antioxidant activity ^7^ |
|  |  |  | 1,5-Anhydro-6-deoxyhexo-2, 3-diulose | C_6_H_8_O_4_ | 144 | 936 | Antioxidant ^8^, antimicrobial ^9^, and anticancer activity ^4^ |
| 16. | 10.60 | 3.80 | 5-Aminouracil | C_4_H_5_N_3_O_2_ | 127 | 963 | Not known |
| 17. | 11.33 | 0.50 | Ethanamine-N-ethyl-N-nitroso | C_4_H_10_N_2_O | 102 | 832 | Not known |
| 18. | 11.54 | 0.44 | 2(3H)-Furanone-5-heptyldihydro | C_11_H_20_O_2_ | 184 | 645 | Not known |
| 19. | 12.78 | 1.03 | 7-Hydroxy-6-methyl-oct-3-enoic acid | C_9_H_16_O_3_ | 172 | 615 | Not known |
| 20. | 12.87 | 2.22 | 4,6-Dimethyl-5-(Nitromethyl)-3-heptanone | C_10_H_19_NO_3_ | 201 | 632 | Not known |
| 21. | 13.25 | 2.85 | 5-Hydroxymethylfurfural | C_6_H_6_O_3_ | 126 | 858 | Antioxidant ^10^, antiproliferative  anti-inflammatory ^11^, antidiabetic ^12^ and anti-hyperuricemic activity |
|  |  |  | 5-(Hydroxymethyl)-2-furaldehyde | C_6_H_6_O_3_ | 126 | 871 | Anticancer ^4^ and antifungal ^6^ activity |
|  |  |  | Thiophene-2-propyl | C_7_H_10_S | 126 | 685 | Anti-inflammatory ^13^, antimicrobial ^9^, antidepressant, and anticonvulsant ^14^ activity |
| 22. | 15.82 | 1.14 | Dodecanoic acid-3-hydroxy | C_12_H_24_O_3_ | 216 | 638 | Antibacterial and antifungal ^9^, anti-apoptotic ^4^, and anti-inflammatory ^13^ activity |
| 23. | 18.51 | 7.99 | Guanosine | C_10_H_13_N_5_O_5_ | 283 | 790 | Antioxidant ^15^, neuromodulator, antidepressant ^16^, and anti-inflammatory ^17^ activity |
| 24. | 20.54 | 0.53 | Desulphosinigrin | C_10_H_17_NO_6_S | 279 | 668 | Antiepileptic, anticancer ^4^, and antimicrobial ^18^ activity |
| 25. | 21.37 | 0.49 |  |  |  | 669 |  |
| 26. | 22.23 | 0.85 | Tetradecanoic acid-ethyl ester | C_16_H_32_O_2_ | 256 | 714 | Anti-inflammatory ^19^, antimicrobial, antioxidant ^20^, and antidiabetic activity and inhibits uric acid production |
|  |  |  | Octadecanoic acid-ethyl ester | C_20_H_40_O_2_ | 312 | 663 | Antimicrobial activity and inhibit uric acid production |
| 27. | 22.64 | 4.78 | Palmitic acid | C_16_H_32_O_2_ | 256 | 773 | Antibacterial and antifungal activity ^21^ |
|  |  |  | Hexadecanoic acid-1-methyl ethyl ester | C_19_H_38_O_2_ | 298 | 683 | Antioxidant activity ^22^ |
| 28. | 22.75 | 3.27 | Mome-inositol | C_7_H_14_O_6_ | 194 | 765 | Antioxidant ^23^, anti-cirrhotic, anti-neuropathic ^24^, antiproliferative ^25^, and anti-hypercholesterolemic activity and lipotropic |
| 29. | 24.99 | 0.60 | Hexadecanoic acid-ethyl ester | C_18_H_36_O_2_ | 284 | 702 | Anti-inflammatory ^19^, anticancer, and antimicrobial ^26^ activity |
| 30. | 25.11 | 0.47 | Eicosanoic acid | C_20_H_40_O_2_ | 312 | 650 | Not known |
| 31. | 25.22 | 0.26 | Estra-1,3,5(10)-trien-17á-ol | C_18_H_24_O | 256 | 702 | Not known |
| 32. | 25.34 | 3.86 | 9-Octadecenoic acid (Z) | C_18_H_34_O_2_ | 282 | 778 | Antioxidant ^27^, antimicrobial ^26^, and anti-inflammatory ^19^ activity and α-glucosidase inhibitor ^28^ |
| 33. | 25.62 | 0.98 | Oleic acid | C_18_H_34_O_2_ | 282 | 794 | Anti-inflammatory ^19^ and antioxidant activity ^27^, α-glucosidase inhibitor ^29^ and improve ß-cell function, endothelial function, and hypothalamic function |
|  |  |  | 9,12-Octadecadienoic acid (Z,Z) | C_18_H_32_O_2_ | 280 | 748 | 5-α reductase inhibitor, anticancer, antioxidant ^30^, anti-inflammatory ^31^, antihyperlipidemic, and antiatherosclerosis activity |
| 34. | 27.54 | 0.50 | Hexadecanoic acid-2,3-dihydroxypropyl ester | C_19_H_38_O_4_ | 330 | 678 | anti-inflammatory ^32^, anticancer, and antimicrobial activity |
| 35. | 27.75 | 0.44 | Cis-5,8,11,14,17-Eicosapentaenoic acid | C_20_H_30_O_2_ | 302 | 698 | Not known |
| 36. | 28.27 | 2.37 | Arachidonic acid | C_20_H_30_O_2_ | 304 | 745 | Necessary for nervous system cell function ^33^ |
| 37. | 31.27 | 0.23 | 6,9,12,15-Docosatetraenoic acid-methyl ester | C_23_H_38_O_2_ | 346 | 694 | Anti-inflammatory ^32^, anti-hypercholesterolemic, anticancer, and hepatoprotective activity |
| 38. | 32.62 | 1.01 | 1,3-Benzenedicarboxylic acid-bis(2-ethylhexyl) ester | C_24_H_38_O_4_ | 390 | 645 | Antibacterial activity |
| 39. | 32.82 | 0.39 | Cholestan-3-OL,2-methylene (3á,5à) | C_28_H_48_O | 400 | 767 | Not known |

**References**

1 Majumder, R., Adhikari, L., Dhara, M. & Sahu, J. Evaluation of anti-inflammatory, analgesic and TNF-alpha inhibition (upon RAW 264.7 cell line) followed by the selection of extract (leaf and stem) with respect to potency to introduce anti-oral-ulcer model obtained from Olax psittacorum (Lam.) Vahl in addition to GC-MS illustration. *J Ethnopharmacol* **263**, 113146, doi:10.1016/j.jep.2020.113146 (2020).

2 Behera, P. & Balaji, S. The forgotten sugar: A review on multifarious applications of melezitose. *Carbohydr Res* **500**, 108248, doi:10.1016/j.carres.2021.108248 (2021).

3 Bindu, T. K. & Udayan, P. S. GC-MS analysis of bioactive compounds in methanolic extract of tubers of Pueraria tuberosa (Roxb. ex Willd.) DC. - Fabaceae. *International Journal of Environment, Agriculture and Biotechnology* **3**, 1493-1498, doi:10.22161/ijeab/3.4.47 (2018).

4 Ududua, U. O., Monanu, M. O. & Chuku, L. C. Proximate Analysis and Phytochemical Profile of Brachystegia eurycoma Leaves. *Asian Journal of Research in Biochemistry* **4**, 1-11, doi:10.9734/ajrb/2019/v4i230064 (2019).

5 Castillo, S., Heredia, N. & Garcia, S. 2(5H)-Furanone, epigallocatechin gallate, and a citric-based disinfectant disturb quorum-sensing activity and reduce motility and biofilm formation of Campylobacter jejuni. *Folia Microbiol (Praha)* **60**, 89-95, doi:10.1007/s12223-014-0344-0 (2015).

6 Jeeva, S. & Krishnamoorthy, A. S. Antifungal Potential of Myco-molecules of Coprinopsis cinerea (Schaeff) S. Gray s. lat. against Fusarium spp. *Madras Agricultural Journal* **105**, 1 (2018).

7 Al-Tai, A. A. & Al-Mayyahi, T. F. A Chemical Study By Using GC-Mass Spectrometry Of The Peel And Seeds Of Punica Granatum L. Plant. *Systematic Reviews in Pharmacy* **12**, 1414-1421 (2021).

8 Upadhyay, P., Shukla, R., Tiwari, K. N., Dubey, G. P. & Mishra, S. K. Neuroprotective effect of Reinwardtia indica against scopolamine induced memory-impairment in rat by attenuating oxidative stress. *Metabolic brain disease* **35**, 1-17, doi:10.1007/s11011-019-00479-0 (2020).

9 Hussein, H. M., Hameed, R. H. & Hameed, I. H. Screening of Bioactive Compounds of Ricinus communis Using GC-MS and FTIR and Evaluation of its Antibacterial and Antifungal Activity. *Indian Journal of Public Health Research & Development* **9**, 467, doi:10.5958/0976-5506.2018.00488.6 (2018).

10 Lee, C.-H. *et al.* Exploring epigallocatechin gallate impregnation to inhibit 5-hydroxymethylfurfural formation and the effect on antioxidant ability of black garlic. *Lwt* **117**, 108628, doi:10.1016/j.lwt.2019.108628 (2020).

11 Kong, F., Fan, C., Yang, Y., Lee, B. H. & Wei, K. 5-hydroxymethylfurfural-embedded poly (vinyl alcohol)/sodium alginate hybrid hydrogels accelerate wound healing. *Int J Biol Macromol* **138**, 933-949, doi:10.1016/j.ijbiomac.2019.07.152 (2019).

12 Ge, Q. *et al.* Network pharmacology-based dissection of the anti-diabetic mechanism of Lobelia chinensis. *Frontiers in pharmacology* **11**, 347, doi:10.3389/fphar.2020.00347 (2020).

13 Altaee, N., Kadhim, M. J. & Hameed, I. H. Characterization of metabolites produced by E. coli and analysis of its chemical compounds using GC-MS. *International Journal of Current Pharmaceutical Review and Research* **7**, 13-19 (2017).

14 Kulandasamy, R., Adhikari, A. V. & Stables, J. P. A new class of anticonvulsants possessing 6 Hz activity: 3,4-dialkyloxy thiophene bishydrazones. *Eur J Med Chem* **44**, 4376-4384, doi:10.1016/j.ejmech.2009.05.026 (2009).

15 Courtes, A. A. *et al.* Guanosine protects against behavioural and mitochondrial bioenergetic alterations after mild traumatic brain injury. *Brain Research Bulletin* **163**, 31-39, doi:10.1016/j.brainresbull.2020.07.003 (2020).

16 Nonose, Y. *et al.* Guanosine enhances glutamate uptake and oxidation, preventing oxidative stress in mouse hippocampal slices submitted to high glutamate levels. *Brain Res* **1748**, 147080, doi:10.1016/j.brainres.2020.147080 (2020).

17 Zizzo, M. G. *et al.* Preventive effects of guanosine on intestinal inflammation in 2, 4-dinitrobenzene sulfonic acid (DNBS)-induced colitis in rats. *Inflammopharmacology* **27**, 349-359, doi:10.1007/s10787-018-0506-9 (2019).

18 Kadhim, M. J., Al-Rubaye, A. F. & Hameed, I. H. Determination of Bioactive Compounds of Methanolic Extract of Vitis vinifera Using GC-MS. *International Journal of Toxicological and Pharmacological Research* **9**, 113-126, doi:10.25258/ijtpr.v9i02.9047 (2017).

19 Pegoraro, N. S. *et al.* Oleic acid-containing semisolid dosage forms exhibit in vivo anti-inflammatory effect via glucocorticoid receptor in a UVB radiation-induced skin inflammation model. *Inflammopharmacology* **28**, 773-786, doi:10.1007/s10787-019-00675-5 (2020).

20 Mohy El-Din, S. M. & Alagawany, N. I. Phytochemical Constituents and Anticoagulation Property of Marine Algae Gelidium crinale, Sargassum hornschuchii and Ulva linza. *Thalassas: An International Journal of Marine Sciences* **35**, 381-397, doi:10.1007/s41208-019-00142-6 (2019).

21 Shokery, E. S., El Ziney, M. G., Yossef, A. H. & Mashaly, R. I. Effect of Green Tea and Moringa Leave Extracts Fortification on the Physicochemical, Rheological, Sensory and Antioxidant Properties of Set-Type Yoghurt. *Advances in Dairy Research* **05**, 1-10, doi:10.4172/2329-888x.1000179 (2017).

22 Kumar, S. S. & Patra, A. Evaluation of phenolic composition, antioxidant, anti-inflammatory and anticancer activities of Polygonatum verticillatum (L.). *J Integr Med* **16**, 273-282, doi:10.1016/j.joim.2018.04.005 (2018).

23 Suganthy, M. & Gajendra, C. V. Chemical characterization of <em>Strychnos</em> <em>nux-vomica</em> L. leaves for biopesticidal properties using GC-MS. *International Journal of Chemical Studies* **8**, 1112-1116, doi:10.22271/chemi.2020.v8.i1o.8398 (2020).

24 Sikri, N., Dhanda, S. & Dalal, S. Kinetics of urease inhibition by different fractions of Cassia fistula. *South African Journal of Botany* **120**, 274-279, doi:10.1016/j.sajb.2018.07.016 (2019).

25 Sogan, N., Kala, S., Kapoor, N. & Nagpal, B. N. Phytochemical analysis of Spergula arvensis and evaluation of its larvicidal activity against malarial vector An.culicfiacies. *South African Journal of Botany* **137**, 351-358, doi:10.1016/j.sajb.2020.11.006 (2021).

26 Alrumman, S. A. In Vitro Antimicrobial Activity and GC–MS Findings of the Gel of Aloe vacillans Forssk. of Abha Region, Saudi Arabia. *Arabian Journal for Science and Engineering* **43**, 155-162, doi:10.1007/s13369-017-2785-7 (2017).

27 Bettadahalli, S., Acharya, P., Ramaiyan, B. & Talahalli, R. R. Evidence on oleic acid and EPA + DHA role in retinal antioxidant defense, leukocyte adhesion, and vascular permeability: Insight from hyperlipidemic rat model. *Journal of Functional Foods* **67**, 103864, doi:10.1016/j.jff.2020.103864 (2020).

28 Yang, D. *et al.* Antioxidant and alpha-Glucosidase Inhibitory Activities Guided Isolation and Identification of Components from Mango Seed Kernel. *Oxid Med Cell Longev* **2020**, 8858578, doi:10.1155/2020/8858578 (2020).

29 Collado-González, J. *et al.* Inhibition of α-glucosidase and α-amylase by Spanish extra virgin olive oils: The involvement of bioactive compounds other than oleuropein and hydroxytyrosol. *Food Chemistry* **235**, 298-307, doi:10.1016/j.foodchem.2017.04.171 (2017).

30 da Rosa, B. V., Sauzem, G. d.-S. & Kuhn, R. C. Obtaining antioxidant compounds from the endophytic fungus Diaporthe schini using heat- and ultrasound-assisted extraction. *Brazilian Journal of Chemical Engineering* **38**, 189-195, doi:10.1007/s43153-021-00089-3 (2021).

31 Murugesan, K., Mulugeta, K., Hailu, E., Tamene, W. & Alagar, Y. S. Insights for integrative medicinal potentials of Ethiopian Kale (Brassica carinata): Investigation of antibacterial, antioxidant potential and phytocompounds composition of its leaves. *Chinese Herbal Medicines* **xxx**, 1-5, doi:10.1016/j.chmed.2020.09.003 (2020).

32 Krupashree, M. K., Renuka, R. & Rajesh, S. Phytochemical investigation of Hybanthus enneaspermus and its cell culture. *Journal of Pharmacognosy and Phytochemistry* **7**, 2847-2851 (2018).

33 Tallima, H. & El Ridi, R. Arachidonic acid: Physiological roles and potential health benefits – A review. *Journal of Advanced Research* **11**, 33-41, doi:10.1016/j.jare.2017.11.004 (2018).


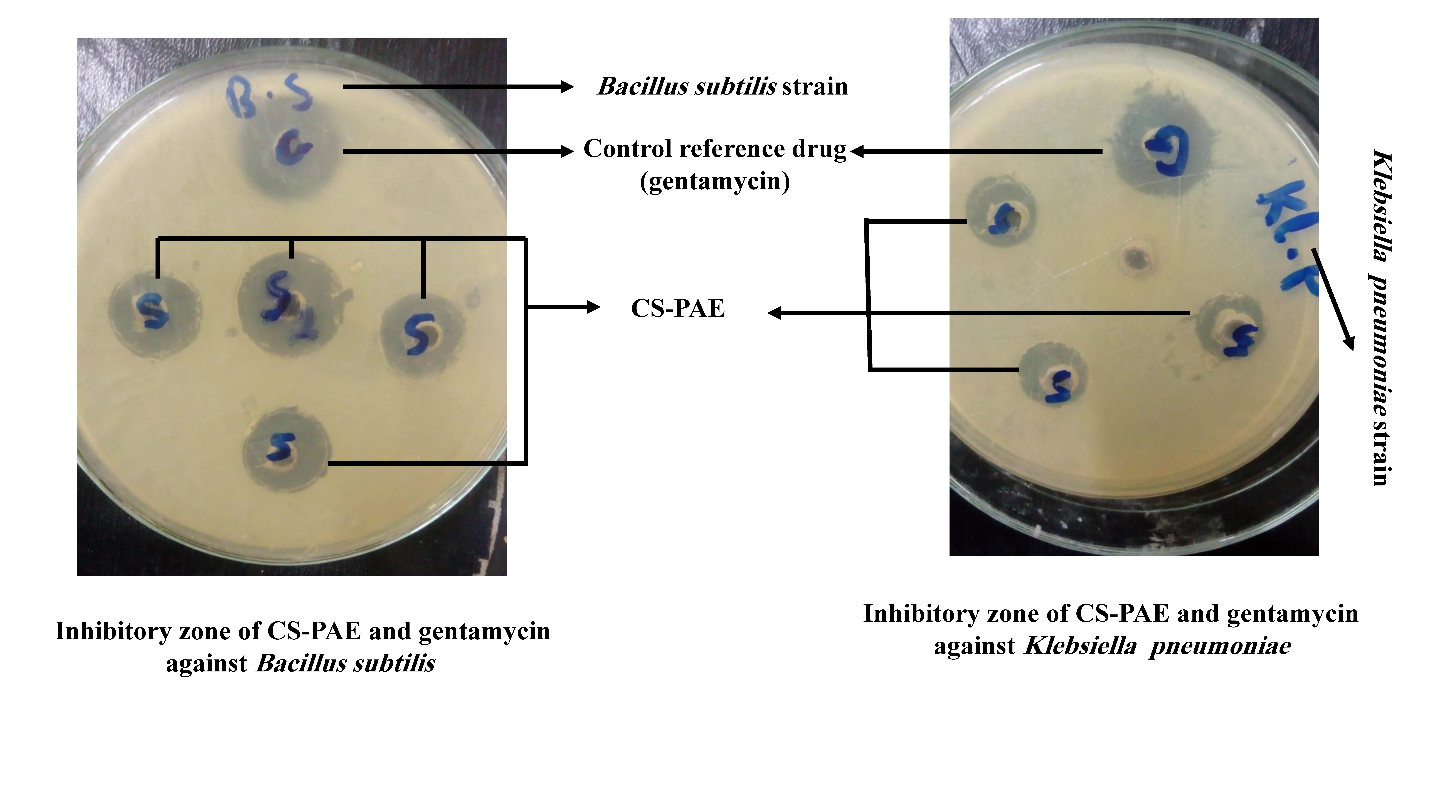


**Fig S1.** Antimicrobial effect of CS-PAE against some bacterial strains.


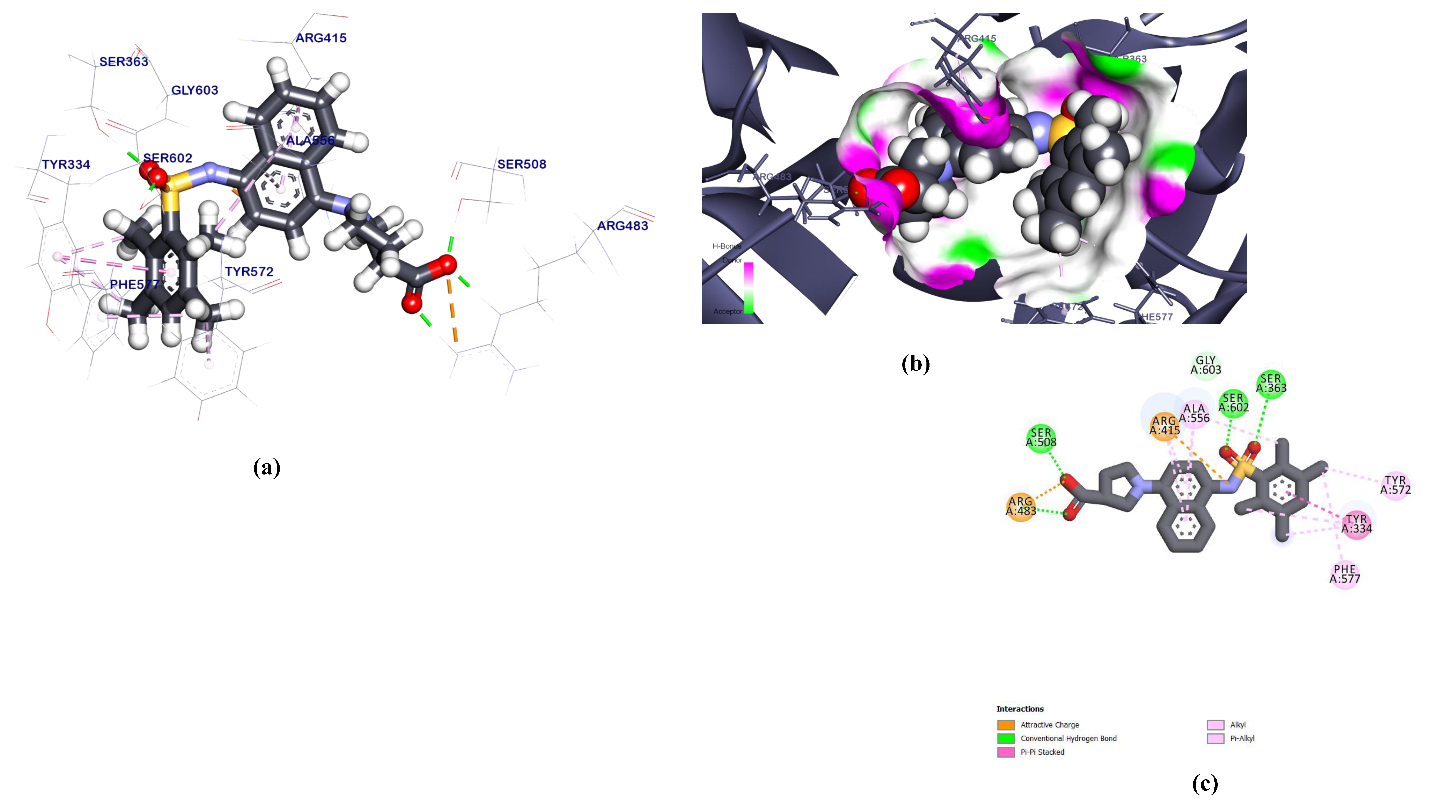


**Fig S2. The Molecular docking representation of RA839 in the binding site of Keap-1/ Nrf2,** **(a)** 3D image of RA839 docked with Keap-1/ Nrf2, **(b)** mapping surface of RA839 occupying the active pocket of Keap-1/ Nrf2, **(c)** 2D image representation of hydrogen bonds (green) and the Pi interactions are represented in purple lines.


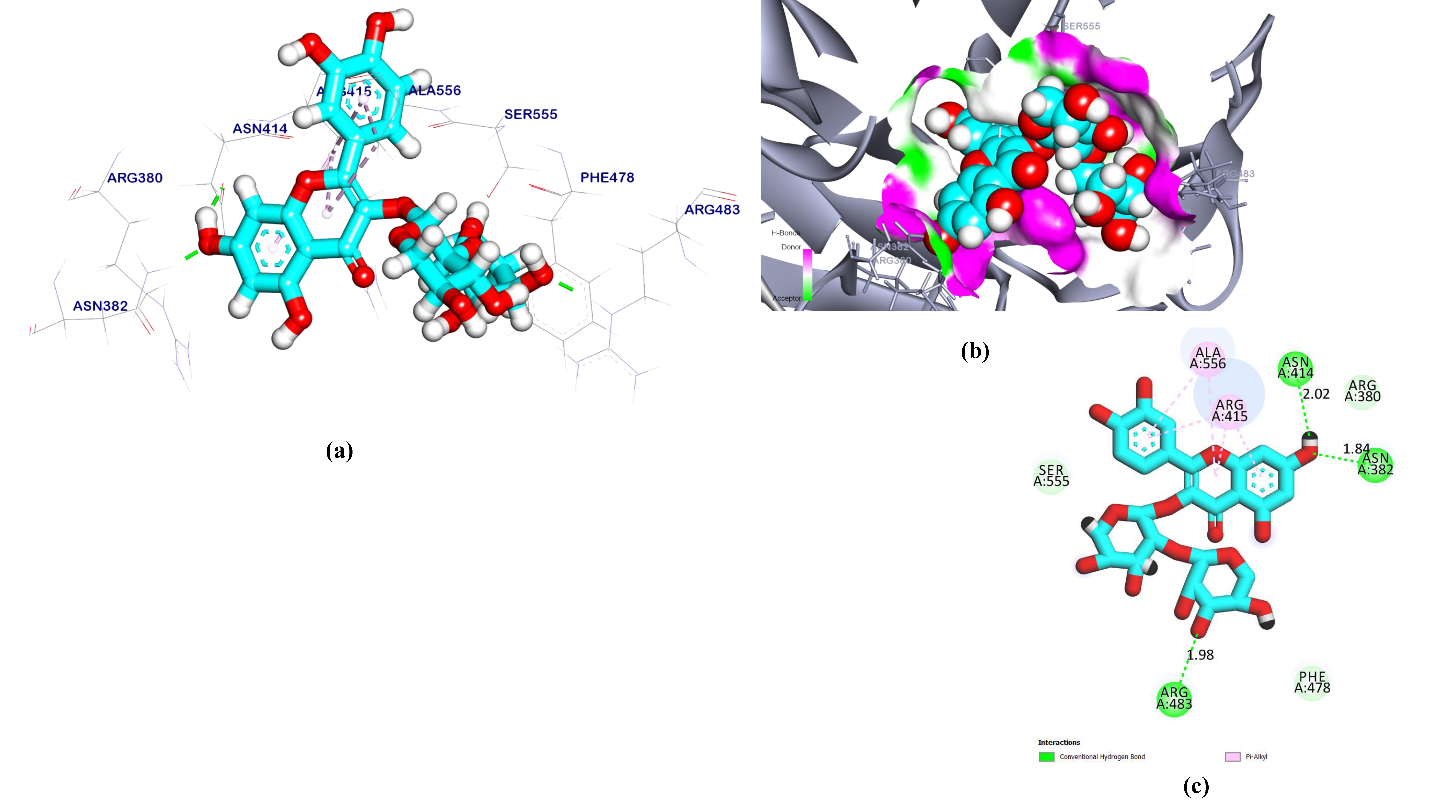


**Fig S3. The Molecular docking representation of quercetin-O-pentoside** **in the binding site of Keap-1/ Nrf2,** **(a)** 3D image of quercetin-O-pentoside docked with Keap-1/ Nrf2, **(b)** mapping surface of quercetin-O-pentoside occupying the active pocket of Keap-1/ Nrf2, **(c)** 2D image representation of hydrogen bonds (green) and the Pi interactions are represented in purple lines.


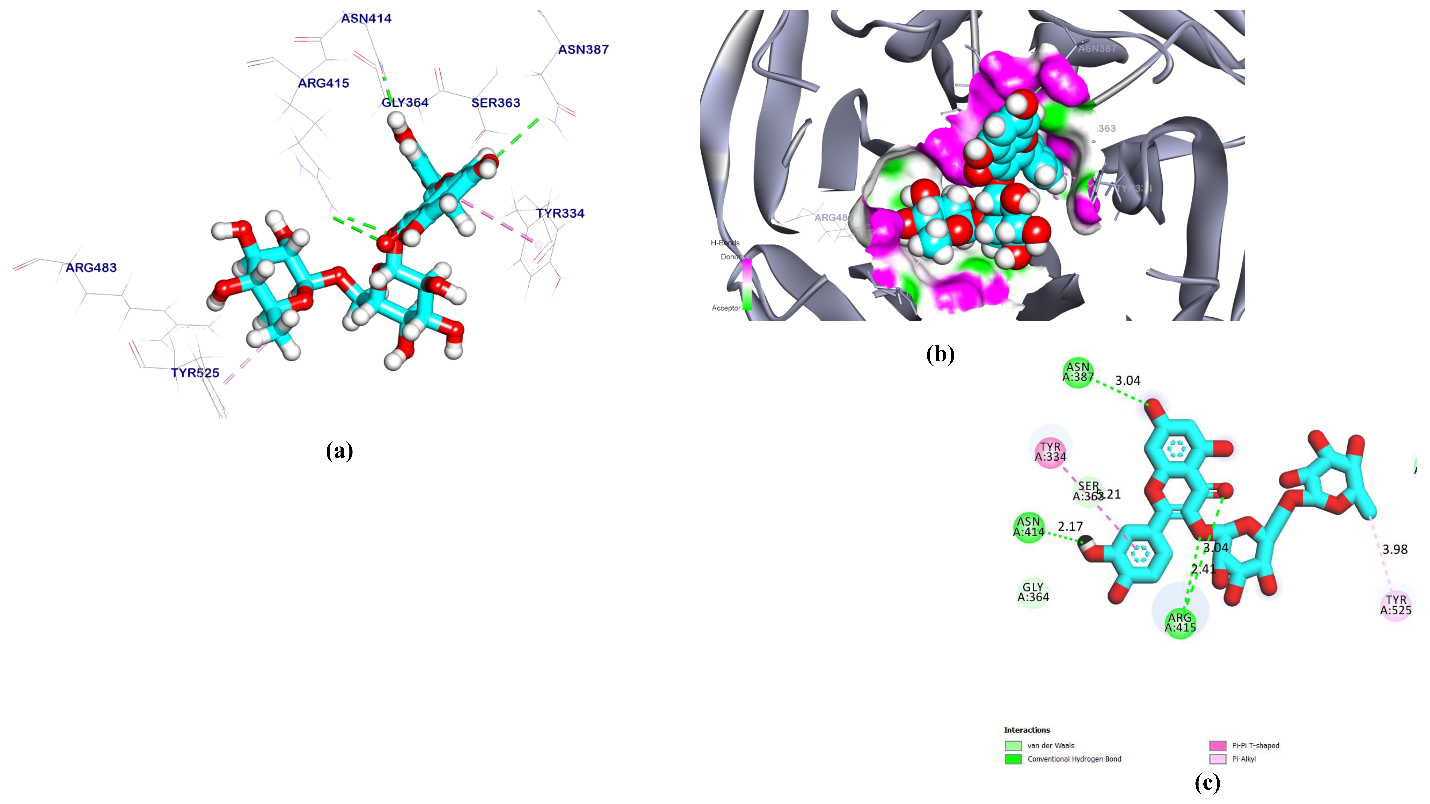


**Fig S4. The Molecular docking representation of rutin in the binding site of Keap-1/ Nrf2,** **(a)** 3D image of rutin docked with Keap-1/ Nrf2, **(b)** mapping surface of rutin occupying the active pocket of Keap-1/ Nrf2, **(c)** 2D image representation of hydrogen bonds (green) and the Pi interactions are represented in purple lines.


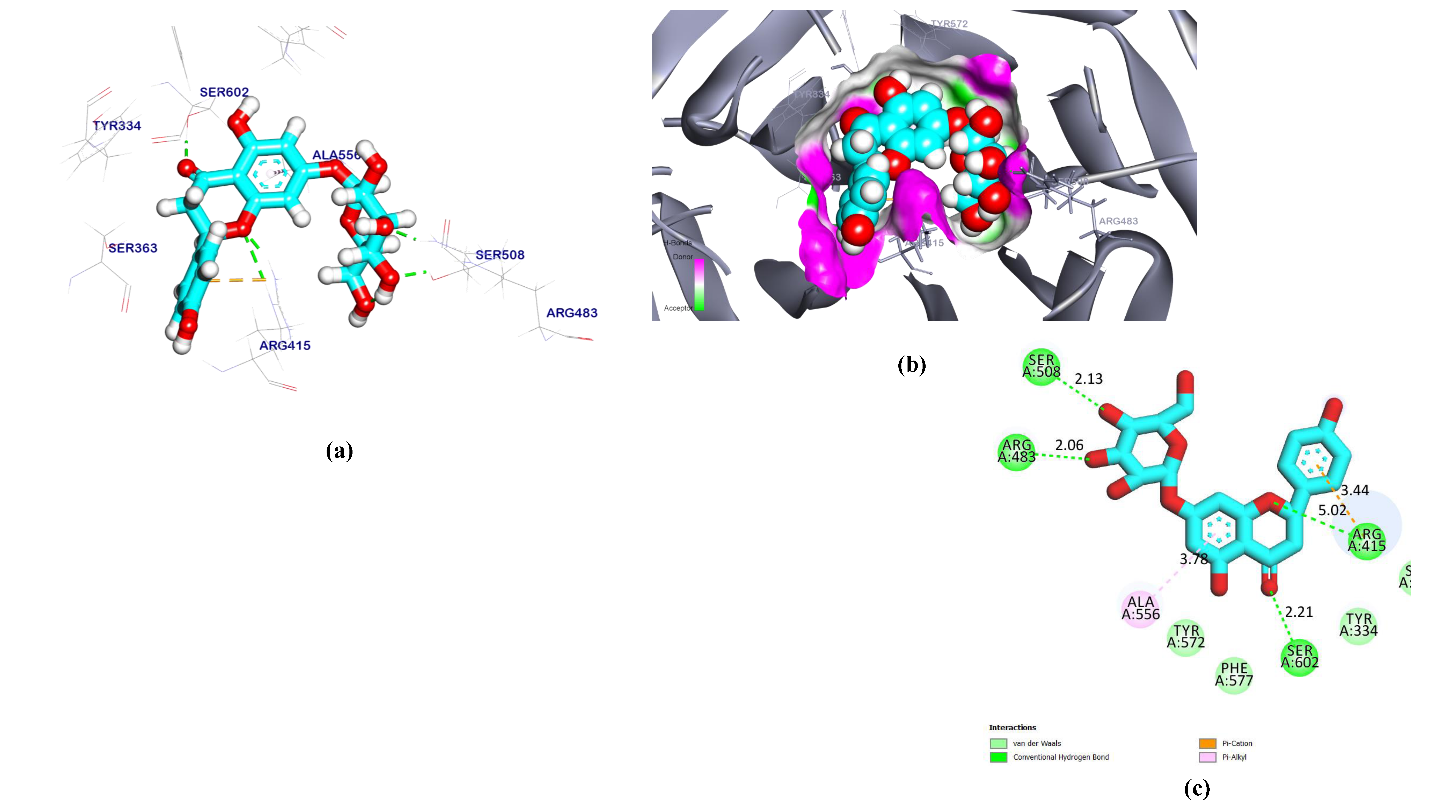


**Fig S5. The Molecular docking representation of naringenin-O-hexoside** **in the binding site of Keap-1/ Nrf2,** **(a)** 3D image of naringenin-O-hexoside docked with Keap-1/ Nrf2, **(b)** mapping surface of naringenin-O-hexoside occupying the active pocket of Keap-1/ Nrf2, **(c)** 2D image representation of hydrogen bonds (green) and the Pi interactions are represented in purple lines.


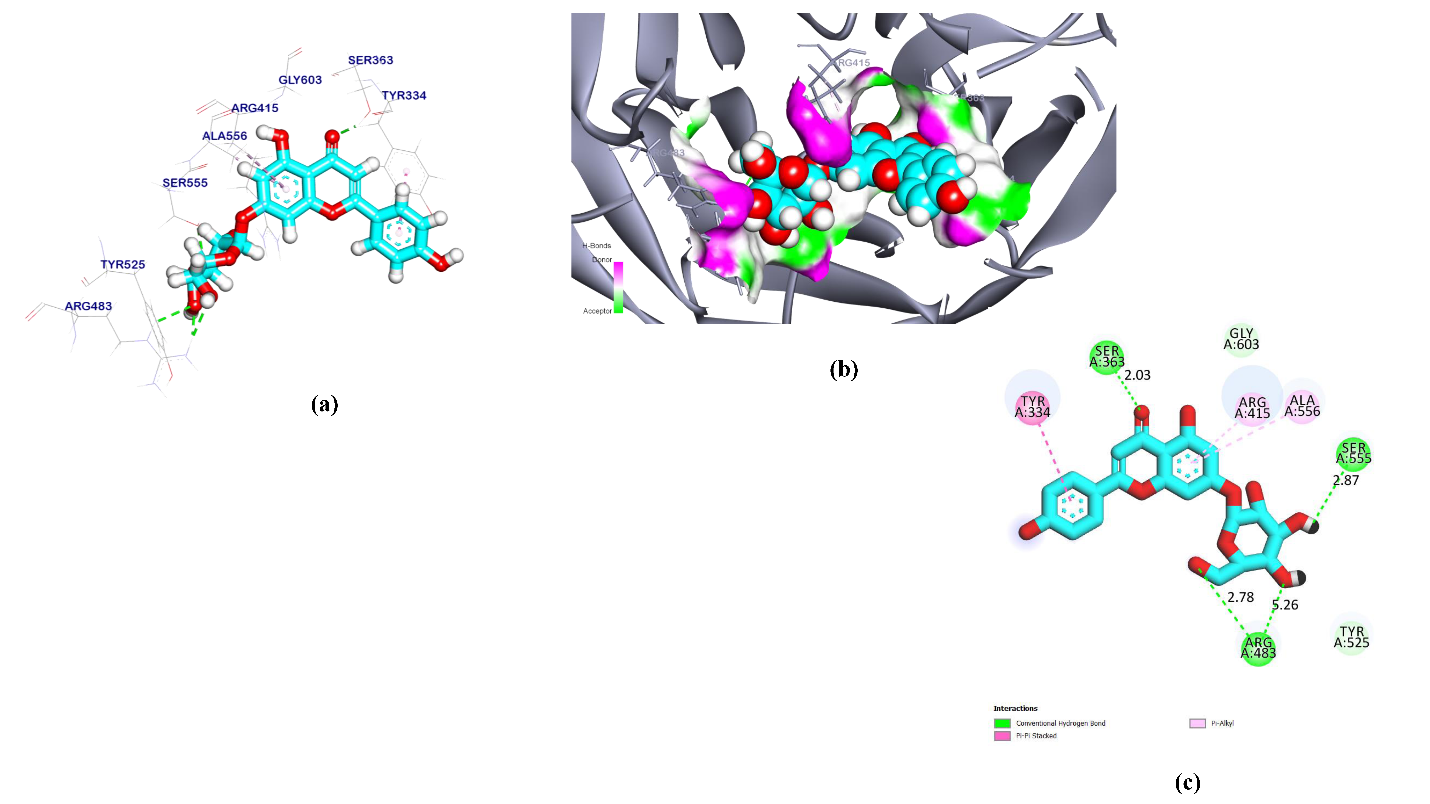


**Fig S6. The Molecular docking representation of apigenin-7-glucoside** **in the binding site of Keap-1/ Nrf2,** **(a)** 3D image of apigenin-7-glucoside docked with Keap-1/ Nrf2, **(b)** mapping surface of apigenin-7-glucoside occupying the active pocket of Keap-1/ Nrf2, **(c)** 2D image representation of hydrogen bonds (green) and the Pi interactions are represented in purple lines.


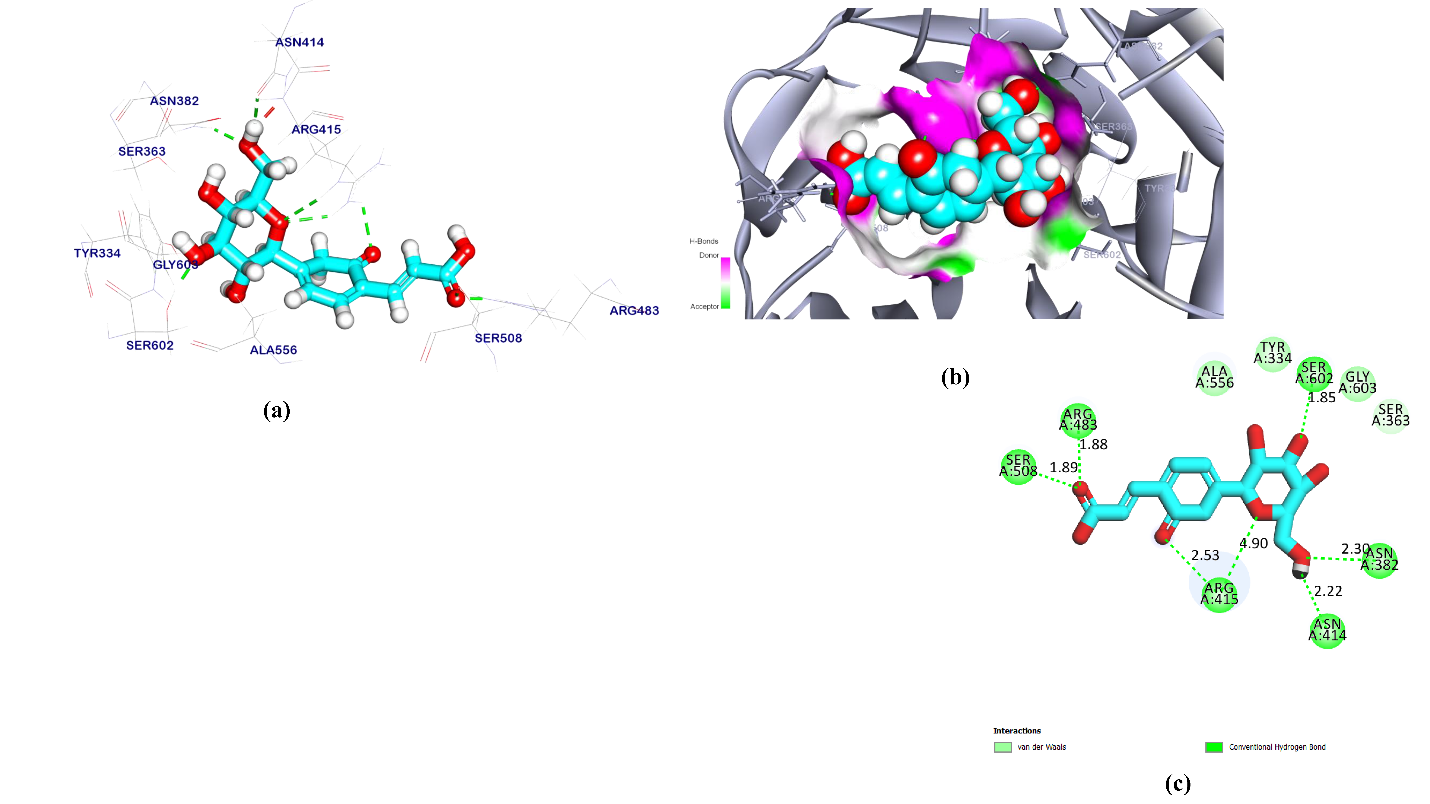


**Fig S7. The Molecular docking representation of *p*-coumaric acid** **in the binding site of Keap-1/ Nrf2,** **(a)** 3D image of *p*-coumaric acid docked with Keap-1/ Nrf2, **(b)** mapping surface of *p*-coumaric acid occupying the active pocket of Keap-1/ Nrf2, **(c)** 2D image representation of hydrogen bonds (green).


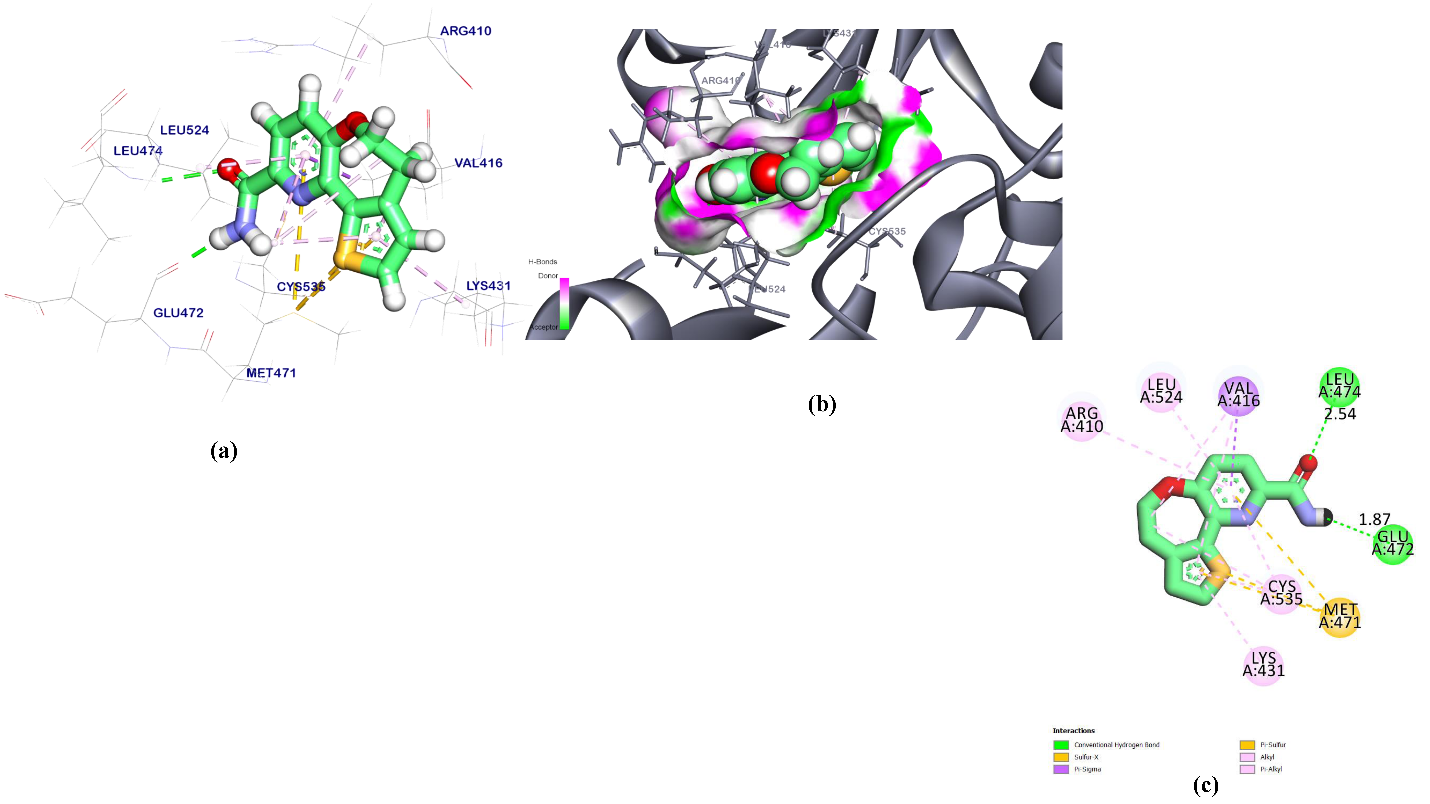


**Fig S8. The Molecular docking representation of benzoxepin** **in the binding site of NF-ĸB,** **(a)** 3D image of benzoxepin docked with NF-ĸB, **(b)** mapping surface of benzoxepin occupying the active pocket of NF-ĸB, **(c)** 2D image representation of hydrogen bonds (green) and the Pi interactions are represented in purple lines.


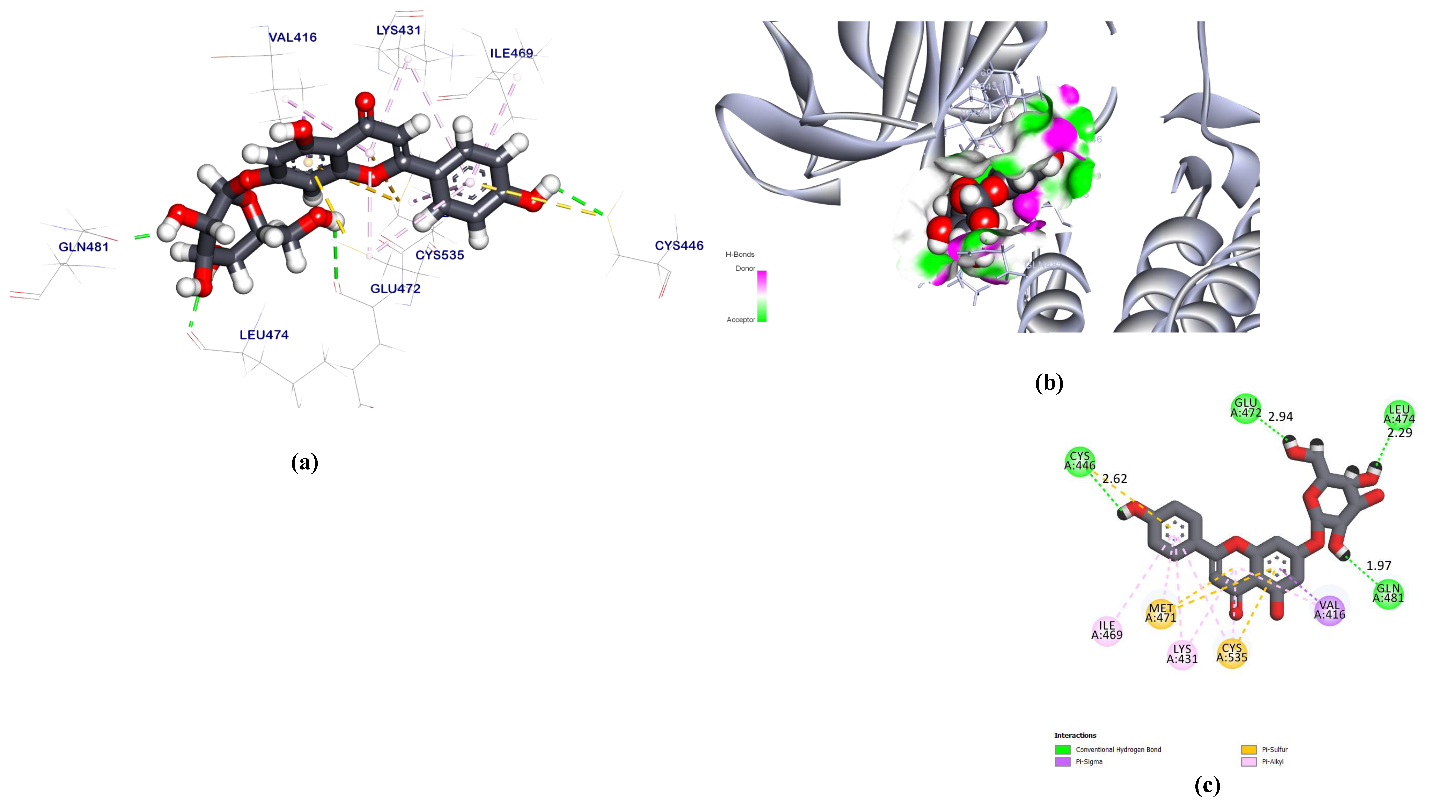


**Fig S9. The Molecular docking representation of apigenin-7-glucoside in the binding site of NF-ĸB,** **(a)** 3D image of apigenin-7-glucoside docked with NF-ĸB, **(b)** mapping surface of apigenin-7-glucoside occupying the active pocket of NF-ĸB, **(c)** 2D image representation of hydrogen bonds (green) and the Pi interactions are represented in purple lines.


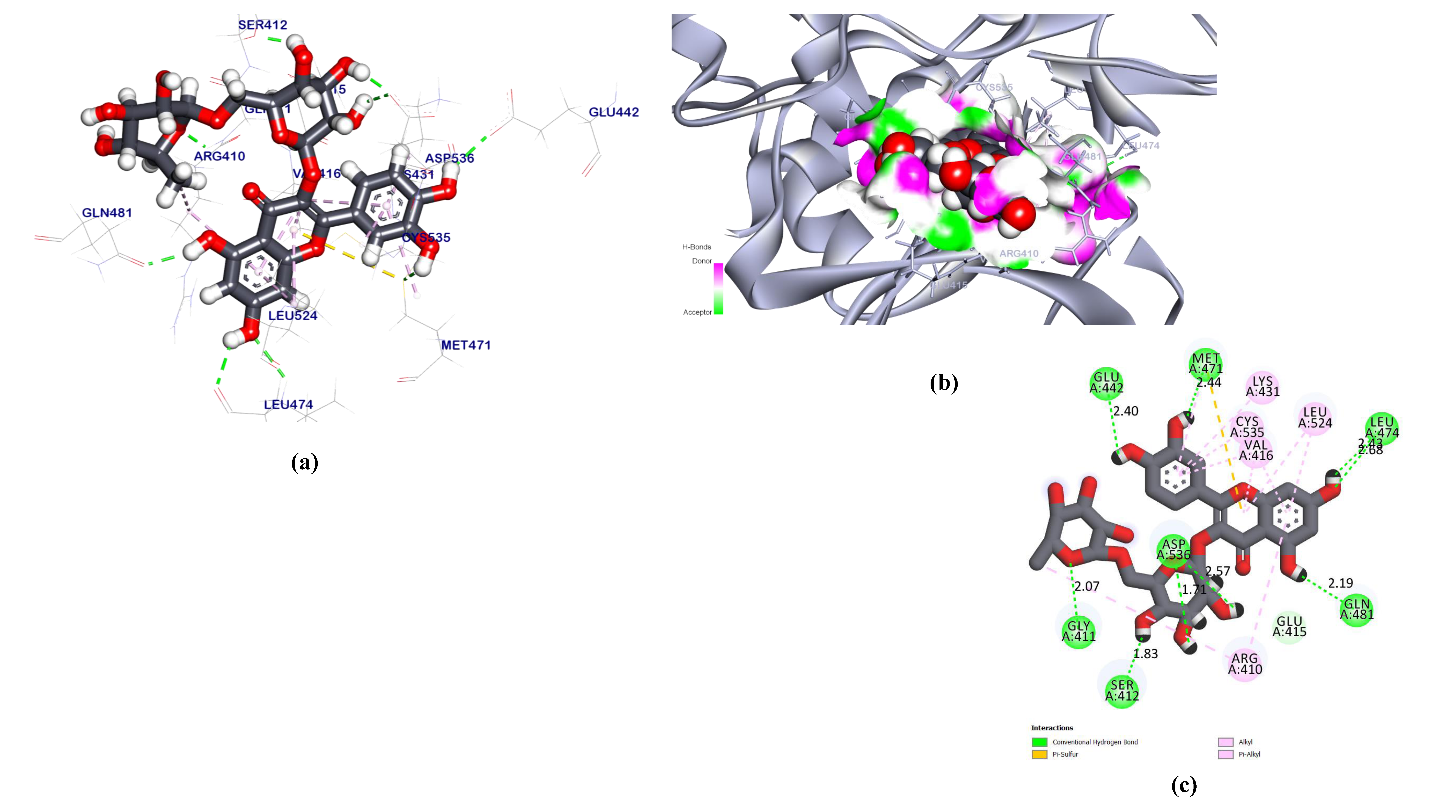


**Fig S10. The Molecular docking representation of rutin** **in the binding site of NF-ĸB,** **(a)** 3D image of rutin docked with NF-ĸB, **(b)** mapping surface of rutin occupying the active pocket of NF-ĸB, **(c)** 2D image representation of hydrogen bonds (green) and the Pi interactions are represented in purple lines.


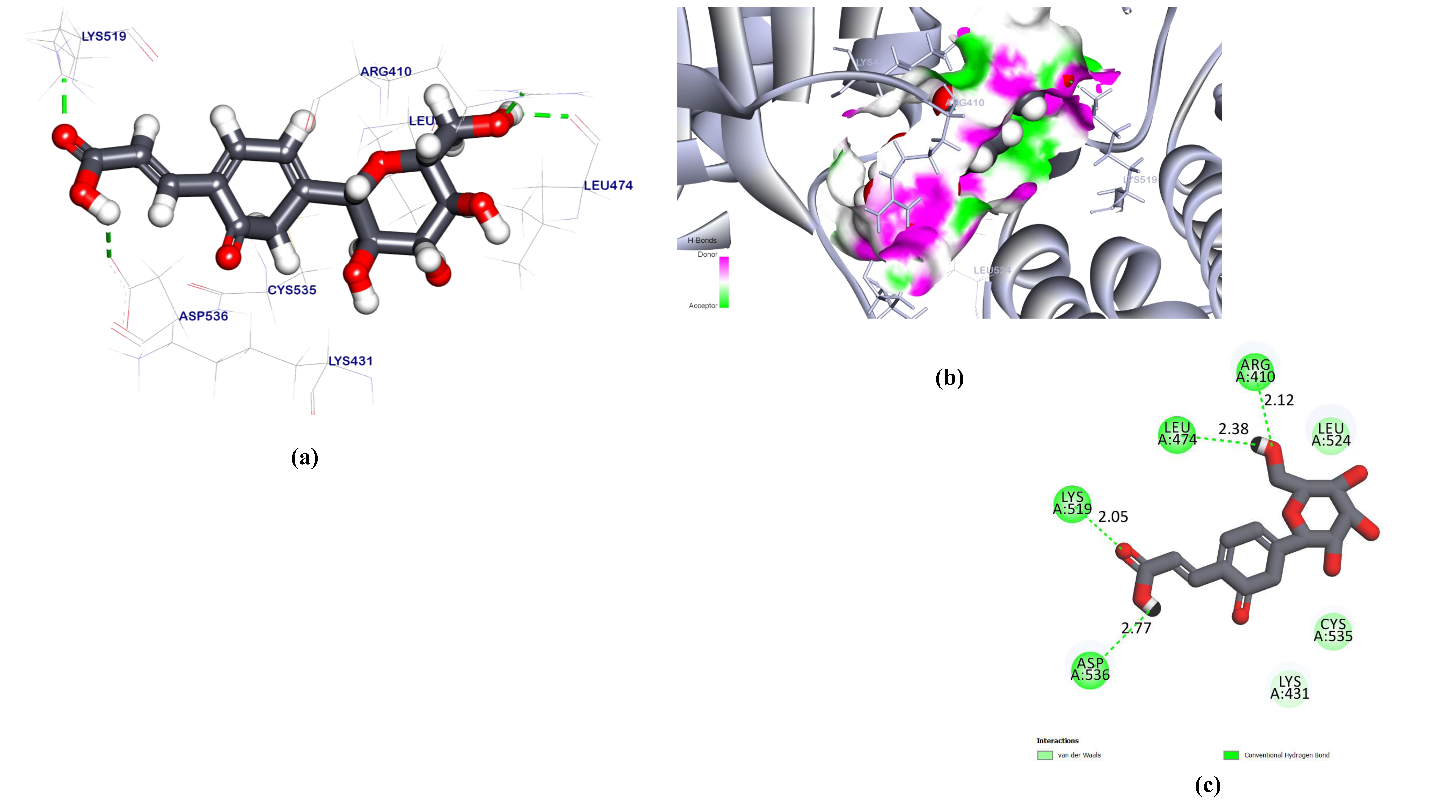


**Fig S11. The Molecular docking representation of *p*-coumaric acid in the binding site of NF-ĸB,** **(a)** 3D image of *p*-coumaric acid docked with NF-ĸB, **(b)** mapping surface of *p*-coumaric acid occupying the active pocket of NF-ĸB, **(c)** 2D image representation of hydrogen bonds (green).


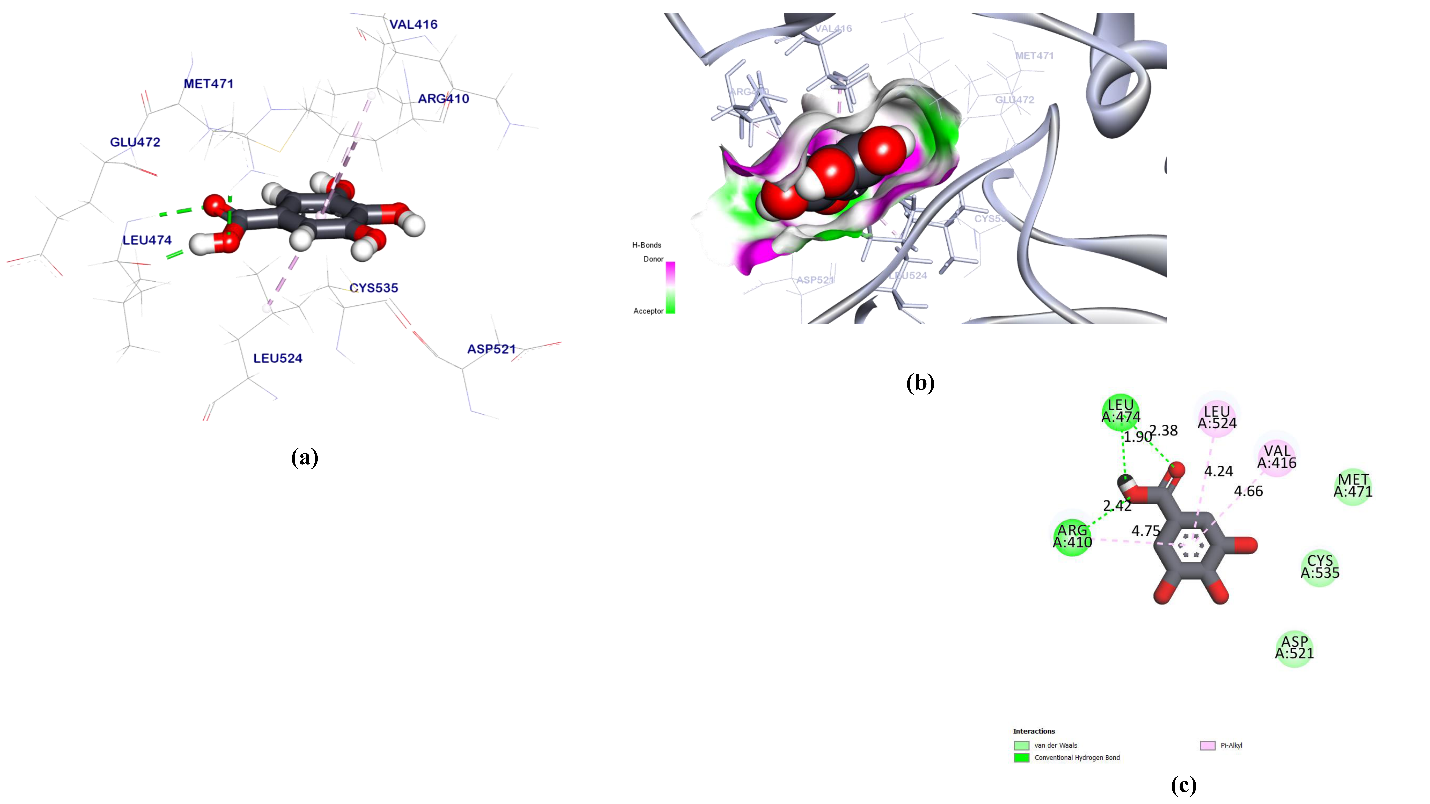


**Fig S12. The Molecular docking representation of gallic acid** **in the binding site of NF-ĸB,** **(a)** 3D image of gallic acid docked with NF-ĸB, **(b)** mapping surface of gallic acid occupying the active pocket of NF-ĸB, **(c)** 2D image representation of hydrogen bonds (green) and the Pi interactions are represented in purple lines.

**Supplementary Figure ligands**

**Fig S1.** Antimicrobial effect of CS-PAE against some bacterial strains.

**Fig S2. The Molecular docking representation of RA839 in the binding site of Keap-1/ Nrf2,** **(a)** 3D image of RA839 docked with Keap-1/ Nrf2, **(b)** mapping surface of RA839 occupying the active pocket of Keap-1/ Nrf2, **(c)** 2D image representation of hydrogen bonds (green) and the Pi interactions are represented in purple lines.

**Fig S3. The Molecular docking representation of quercetin-O-pentoside** **in the binding site of Keap-1/ Nrf2,** **(a)** 3D image of quercetin-O-pentoside docked with Keap-1/ Nrf2, **(b)** mapping surface of quercetin-O-pentoside occupying the active pocket of Keap-1/ Nrf2, **(c)** 2D image representation of hydrogen bonds (green) and the Pi interactions are represented in purple lines.

**Fig S4. The Molecular docking representation of rutin in the binding site of Keap-1/ Nrf2,** **(a)** 3D image of rutin docked with Keap-1/ Nrf2, **(b)** mapping surface of rutin occupying the active pocket of Keap-1/ Nrf2, **(c)** 2D image representation of hydrogen bonds (green) and the Pi interactions are represented in purple lines.

**Fig S5. The Molecular docking representation of naringenin-O-hexoside** **in the binding site of Keap-1/ Nrf2,** **(a)** 3D image of naringenin-O-hexoside docked with Keap-1/ Nrf2, **(b)** mapping surface of naringenin-O-hexoside occupying the active pocket of Keap-1/ Nrf2, **(c)** 2D image representation of hydrogen bonds (green) and the Pi interactions are represented in purple lines.

**Fig S6. The Molecular docking representation of apigenin-7-glucoside** **in the binding site of Keap-1/ Nrf2,** **(a)** 3D image of apigenin-7-glucoside docked with Keap-1/ Nrf2, **(b)** mapping surface of apigenin-7-glucoside occupying the active pocket of Keap-1/ Nrf2, **(c)** 2D image representation of hydrogen bonds (green) and the Pi interactions are represented in purple lines.

**Fig S7. The Molecular docking representation of *p*-coumaric acid** **in the binding site of Keap-1/ Nrf2,** **(a)** 3D image of *p*-coumaric acid docked with Keap-1/ Nrf2, **(b)** mapping surface of *p*-coumaric acid occupying the active pocket of Keap-1/ Nrf2, **(c)** 2D image representation of hydrogen bonds (green).

**Fig S8. The Molecular docking representation of benzoxepin** **in the binding site of NF-ĸB,** **(a)** 3D image of benzoxepin docked with NF-ĸB, **(b)** mapping surface of benzoxepin occupying the active pocket of NF-ĸB, **(c)** 2D image representation of hydrogen bonds (green) and the Pi interactions are represented in purple lines.

**Fig S9. The Molecular docking representation of apigenin-7-glucoside in the binding site of NF-ĸB,** **(a)** 3D image of apigenin-7-glucoside docked with NF-ĸB, **(b)** mapping surface of apigenin-7-glucoside occupying the active pocket of NF-ĸB, **(c)** 2D image representation of hydrogen bonds (green) and the Pi interactions are represented in purple lines.

**Fig S10. The Molecular docking representation of rutin** **in the binding site of NF-ĸB,** **(a)** 3D image of rutin docked with NF-ĸB, **(b)** mapping surface of rutin occupying the active pocket of NF-ĸB, **(c)** 2D image representation of hydrogen bonds (green) and the Pi interactions are represented in purple lines.

**Fig S11. The Molecular docking representation of *p*-coumaric acid in the binding site of NF-ĸB,** **(a)** 3D image of *p*-coumaric acid docked with NF-ĸB, **(b)** mapping surface of *p*-coumaric acid occupying the active pocket of NF-ĸB, **(c)** 2D image representation of hydrogen bonds (green).

**Fig S12. The Molecular docking representation of gallic acid** **in the binding site of NF-ĸB,** **(a)** 3D image of gallic acid docked with NF-ĸB, **(b)** mapping surface of gallic acid occupying the active pocket of NF-ĸB, **(c)** 2D image representation of hydrogen bonds (green) and the Pi interactions are represented in purple lines.

**Supplementary Table ligands**

**Table S1.** Compounds detected in GC-MS analysis of CS-PAE.
